# High‐Performance Piezoresistive Sensors via Pd/Sn‐Free Graphene‐Catalyzed Metallization of Thermally Repairable Microfiber Scaffolds

**DOI:** 10.1002/advs.77794

**Published:** 2026-09-16

**Authors:** ByungJun Kim, Su Bin Choi, Yeonwook Jeong, Jeong‐Won Yoon, Seung‐Boo Jung, Jong‐Woong Kim

**Affiliations:** ^1^ Department of Semiconductor Convergence Engineering Sungkyunkwan University Suwon Republic of Korea; ^2^ Department of Electrical and Computer Engineering University of Virginia Charlottesville Virginia USA; ^3^ Department of Electrical and Electronic Engineering Yonsei University Seoul Republic of Korea; ^4^ Department of Advanced Materials Engineering Chungbuk National University Cheongju Republic of Korea; ^5^ School of Advanced Materials Science & Engineering Sungkyunkwan University Suwon Republic of Korea; ^6^ School of Mechanical Engineering Sungkyunkwan University Suwon Republic of Korea

**Keywords:** conductive microfiber, electroless plating, graphene, pressure sensor, thermally assisted recovery

## Abstract

A Pd/Sn‐free, thermally repairable piezoresistive pressure sensor is reported based on electrospun poly(ε‐caprolactone) microfibers metallized through a graphene‐mediated electroless copper process. The conformal graphene interlayer provides catalytic defect sites and electron‐rich domains for uniform Cu nucleation while reinforcing the Cu–polymer interface, yielding a porous PCL/graphene/Cu core–shell scaffold with robust electrical and mechanical integrity. The multilayer sensor exhibits a sensitivity of 280.9 kPa^−1^ over 0.098–200 kPa with good linearity (R^2^ = 0.905), a response/recovery time of 69.3/69.2 ms, and stable operation over 100 000 loading cycles at 100 kPa. The metallized scaffold retains fiber morphology and sensing capability after exposure to 180°C, while damage recovery is enabled by thermally induced softening and reconnection of the PCL core at 80°C. After repeated cut‐and‐repair cycles, the device restores > 95% conductivity and > 90% mechanical strength within 15 min. A 6 × 6 sensor array further enables spatial mapping of loads as low as 1 g with minimal crosstalk, highlighting a practical platform for repairable wearable and tactile sensing.

## Graphical abstract

The multilayer fiber‐based PGC sensor, fabricated via graphene‐mediated electroless copper deposition, demonstrates high sensitivity, mechanical durability, and fast response speed. Additionally, it features low‐temperature thermal assisted recovery and thermal resistance, providing a promising platform for wearable electronic devices and IoT applications.

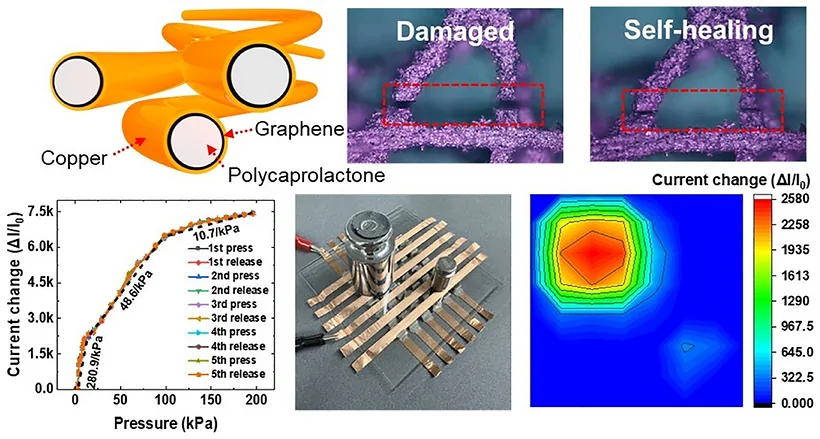

## 1 Introduction

Pressure sensors have become indispensable components in modern technology, finding extensive applications across a broad range of industries including wearable electronics, the Internet of Things (IoT), environmental monitoring, robotics, healthcare, and automotive engineering [1, 2, 3]. These devices play a crucial role in detecting environmental fluctuations, identifying physical interactions, monitoring structural integrity, and tracking biomedical signals, thereby facilitating advancements in smart infrastructure, precision medicine, and autonomous systems [4, 5]. To meet the stringent demands of these emerging fields, next‐generation pressure sensors must exhibit high sensitivity, a wide pressure detection range spanning from ultralow to high pressures, rapid response times, and mechanical robustness with stable long‐term performance to ensure operational reliability [6, 7, 8].

Piezoresistive sensors, valued for low power consumption, broad dynamic range, fast response, and high sensitivity, are widely used in health‐monitoring and pressure‐sensing systems to monitor critical physical and physiological parameters like respiratory and blood pressure with precision [9, 10, 11, 12]. Despite these advantages, achieving simultaneous optimization of sensitivity, sensing range, and mechanical reliability remains challenging, limiting their deployment in demanding applications [12]. Among these, conductive fiber‐based pressure sensors, which exploit inter‐fiber contact for conductivity changes, are promising but often face trade‐offs due to network saturation at high pressures, structural deformation under cyclic loading, and degradation of weak surface coatings from poor interfacial adhesion [13, 14, 15]. Conventional fabrication methods, relying on surface‐coated polymers or nanomaterials, suffer from inadequate conductivity and long‐term instability [16, 17]. Developing fiber‐based sensors that integrate high sensitivity, broad range, durability, and thermally repairable functionality based on damage‐tolerant functional recovery remains a significant challenge.

Recent research has concentrated on integrating novel nanomaterials—namely MXenes, graphene, and carbon nanotubes (CNTs)—into electrospun fiber‐based architectures to enhance pressure sensing performance [18, 19, 20, 21, 22]. For instance, Ren et al. reported an electrospun MXene/polyvinyl pyrrolidone (PVP) nanofiber membrane with moderate sensitivity (∼0.5 kPa^−1^) and a fast response (∼45 ms); however, MXene aggregation and surface oxidation limited long‐term stability [18]. Liu et al. developed an electrospun polyurethane (PU)/multiwalled carbon nanotube (MWCNT) fiber mat functionalized with PEDOT, achieving wide‐range detection and durability (∼18 000 cycles); nonetheless, PEDOT–CNT layer interfacial delamination under cyclic load remained problematic [20]. Bi et al. introduced oriented thermoplastic polyurethane/polyacrylonitrile (TPU/PAN) nanofibers coated with MXene, obtaining reasonable sensitivity (6.7 kPa^−1^) across a large pressure span; yet, oriented electrospinning hampered scalability [21]. Furthermore, Mahmoud et al. fabricated graphene‐dispersed polyvinyl acetate (PVAc) electrospun nanofibers for piezocapacitive sensors, which displayed improved sensitivity but faced challenges in maintaining uniform graphene dispersion over large areas [22]. These fiber‐based designs demonstrate significant advancements, yet each continues to confront limitations in nanomaterial stability, interface resilience, alignment uniformity, or manufacturing scalability, which must be addressed to enable reliable, multifunctional pressure sensors for real‐world applications.

To further improve durability and longevity, various repairable conductive fibers incorporating dynamic covalent bonds or reversible crosslinking chemistries, such as Diels–Alder reactions, have been introduced [23, 24]. These systems rely on phase transitions under specific thermal or mechanical stimuli, offering repair capabilities but inherently compromising stability under uncontrolled environmental conditions. As a result, they frequently suffer from thermal instability, environmental vulnerability, and mechanical performance deterioration under cyclic loading. Alternatively, high‐strength conductive fibers can enhance robustness, but typically do so at the expense of sensitivity to low‐pressure stimuli [25]. Therefore, a multifunctional sensor platform is required that simultaneously provides high sensitivity, a wide sensing range, mechanical integrity, and damage‐tolerant functional recovery. In this system, heat‐assisted repair is enabled by the thermoplastic PCL matrix, while the graphene/Cu interfacial design provides conductive and structural functionality that cannot be achieved by the polymer alone.

In this study, we propose a microfiber network‐based pressure sensor that concurrently satisfies these critical requirements, with a primary focus on a multilayer fabrication methodology: using of graphene as a mediated interfacial layer for electroless copper plating on electrospun polymer fibers. Specifically, electrospun poly(ε‐caprolactone) (PCL) microfibers were employed as the structural foundation, taking advantage of their low melting temperature (∼60°C) to enable thermally assisted repair as a complementary feature. At the same time, the conformal graphene/Cu shell is designed to preserve the overall fiber morphology and conductive framework at elevated temperatures, thereby distinguishing low‐temperature repairability from high‐temperature structural stability. These fibers were subsequently coated with graphene and a copper layer via electroless deposition, resulting in a robust and conductive PCL/graphene/Cu (PGC) core–shell architecture. The incorporation of a graphene interlayer represents the key design innovation, vital to both the fabrication process and sensor performance. Direct electroless deposition of copper onto pristine PCL fibers is fundamentally limited by three challenges: (1) the inert nature of the polymer surface impedes effective Cu deposition, (2) poor interfacial adhesion between copper and the polymer reduces structural stability, and (3) the polymer network is prone to damage under harsh chemical activation conditions. Graphene overcomes these limitations by serving as a chemically and structurally conformal, and conductive seeding layer [26]. It provides uniform nucleation sites for homogeneous and continuous copper film growth through its defect‐containing graphitic structure and chemically active surface characteristics—derived from defect sites and electron‐rich domains that facilitate Cu ion reduction—thereby eliminating the island‐like morphology often observed with traditional Pd/Sn‐based activation and avoiding the use of toxic catalysts. Additionally, the atomic thinness and flexibility of graphene allow it to intimately conform to the PCL surface, forming strong van der Waals interactions that enhance adhesion and prevent delamination. This hierarchical PGC configuration ensures robust mechanical integrity even under repeated mechanical deformation. Importantly, the graphene coating process is conducted under mild conditions, thereby preserving the delicate morphology of the electrospun fibers and maintaining the porous microstructure essential for high‐performance sensing [27]. PCL, the main material of the sensor, acts as a thermoplastic matrix responsible for thermal recovery, while graphene coated on the fiber surface helps stabilize the interfacial structure and conduction pathways during the recovery process. The Cu layer deposited on the graphene‐coated fiber primarily provides conductivity and pressure sensing functions. The resulting sensor represents a unique combination of characteristics achieved simultaneously: (1) wide range of pressure sensing using multilayer structures; (2) restoration of sensing capabilities through recombination of damaged sites; and (3) high temperature structural stability and high sensing sensitivity enabled by graphene/Cu shells. These synergistic features significantly distinguish our approach from prior designs based on nanocomposites or multilayer fiber configurations, primarily because the graphene‐mediated Cu plating provides superior conductivity, adhesion, and structural integrity without compromising fiber porosity or flexibility. This work presents a scalable and practical strategy for the development of high‐performance fiber‐based pressure sensors. Through systematic material selection and hierarchical structural design, this study provides valuable insights into next‐generation sensor platforms with multifunctional performance attributes suitable for repairable and reusable wearable, flexible sensing, and industrial applications.

## 2 Results and Discussion

**FIGURE 1:**
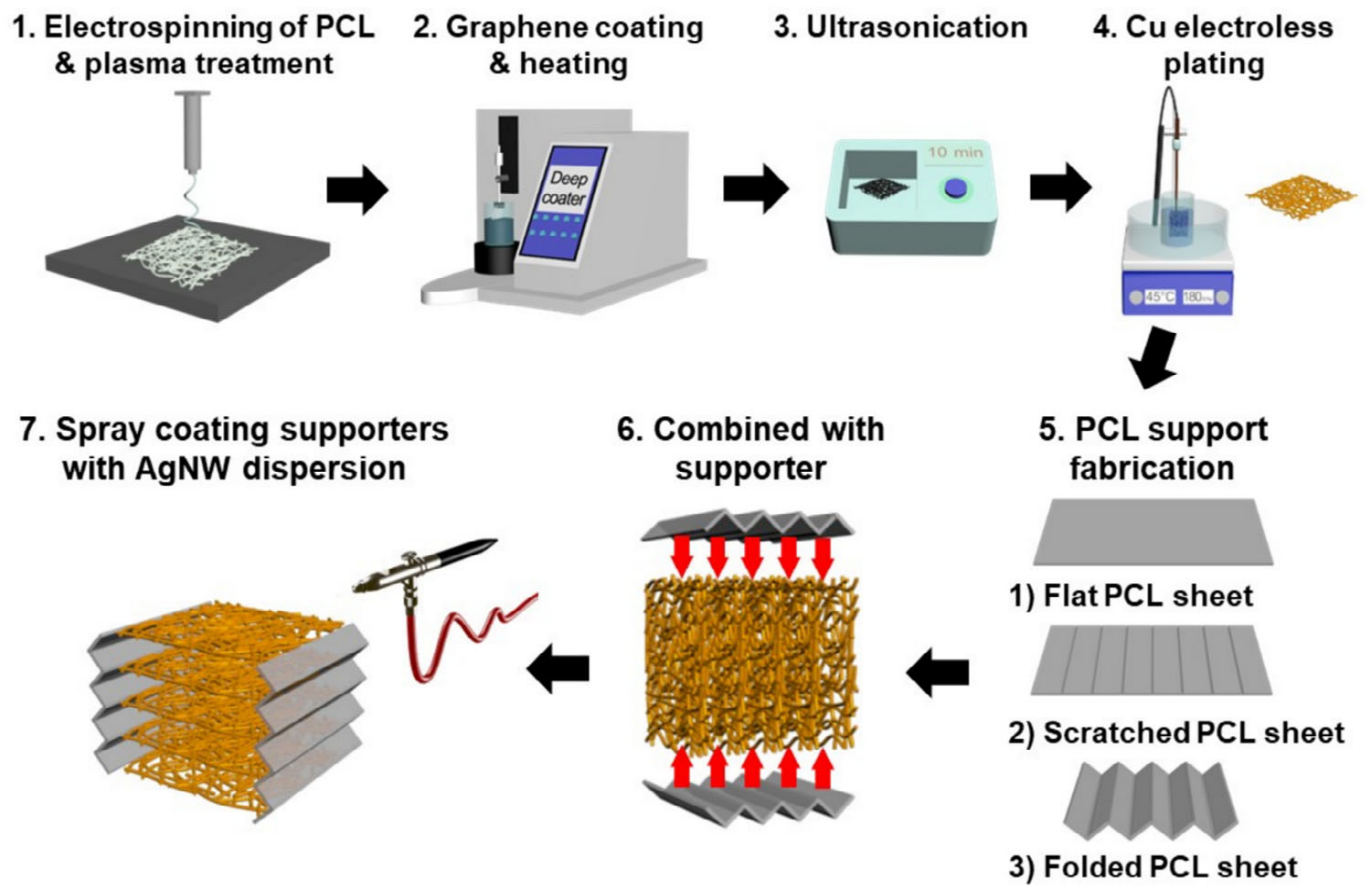
Schematic illustration of the layer by layer assembly of the PGC conductive microfiber network pressure sensor. The diagram highlights (1) electrospinning of PCL microfibers and atmospheric plasma activation to introduce surface functional groups, (2) sequential dip coating in graphene dispersion with intermediate drying and ultrasonic cleaning steps, and (3) conformal electroless copper plating to form a continuous metallic shell, followed by stacking onto a PCL support and silver nanowire (AgNW) electrode deposition.

Figure 1 presents a detailed schematic illustration of the sensor fabrication process. Initially, a nonwoven network of randomly oriented PCL microfibers was produced via electrospinning. To reinforce mechanical robustness and establish a conductive interfacial layer, the as‐spun mat underwent atmospheric‐pressure plasma activation, introducing polar ─OH and ─COOH groups that enhanced subsequent graphene flake adhesion. The plasma‐treated mat was then dip‐coated in a graphene dispersion, where van der Waals interactions and hydrogen bonding enabled uniform physical and chemical adsorption of graphene on the fiber surfaces. After each coating cycle, the mat was air‐dried to evaporate the solvent, and this dip‐and‐dry sequence was repeated until homogeneous coverage was achieved, as confirmed by optical microscopy. A thermal annealing step at 80°C for 10 min followed, during which thermal softening and limited local flow of the PCL fibers promoted strong interfacial binding with the graphene flakes. Subsequent cooling at room temperature induced slight fiber contraction, further increasing contact area and strengthening the composite interface. Loose graphene residues were removed by ultrasonic cleaning in deionized water, and the mat was dried at 40°C prior to metal deposition.

Next, electroless copper plating was performed at 45°C for 3–4.5 h in a CuSO_4_‐based bath. The conductive surface of the defective graphene‐coated fibers provided an interfacial environment favorable for Cu deposition, enabling uniform nucleation and the formation of a tight copper shell without an additional activation step, thereby creating a PGC microfiber network. To construct a highly sensitive pressure sensor, five PGC layers were assembled onto a spin–coated PCL support. The support was fabricated by spin‐coating a low‐concentration PCL solution, thermal curing to introduce linear defects, and folding into a zigzag architecture to localize strain under compression. PGC patches (10 × 10 mm) were bonded using low‐viscosity PCL solution, which solidified upon solvent evaporation to form a cohesive five‐layer structure. Figures S1 and S2 provide visual details of the PCL substrate spin‐coating and linear defect introduction, respectively, while Figure S3 illustrates the resulting low‐resistance, five‐layer assembly. Finally, the support was spray‐coated with 0.3 wt.% AgNW dispersion to establish low‐resistance electrodes. Optimization of AgNW loading (0.1–0.5 wt.%) demonstrated: (i) uniform deposition on the PCL support up to 0.3 wt.% before onset of aggregation (Figure S4); (ii) enhanced surface coverage and nanowire network formation at 0.3 wt.%, confirmed by enlarged microscopy images (Figure S5); and (iii) a decrease in sheet resistance from ∼10^6^ Ω at 0.1 wt.% to ∼10^3^ Ω at 0.3 wt.%, with further loading offering marginal gains (Figure S6). These results identify 0.3 wt% AgNW as the optimum concentration for balancing conductivity and structural integrity. The graphene and electroless Cu‐plated multilayer design coupled to the fiber surface induces the sensor's ultrahigh sensitivity and wide pressure sensing range. The thermoplastic response of PCL serves as the primary recovery mechanism under thermal treatment, whereas graphene/Cu mainly provides interfacial stabilization and conductive functionality.

**FIGURE 2:**
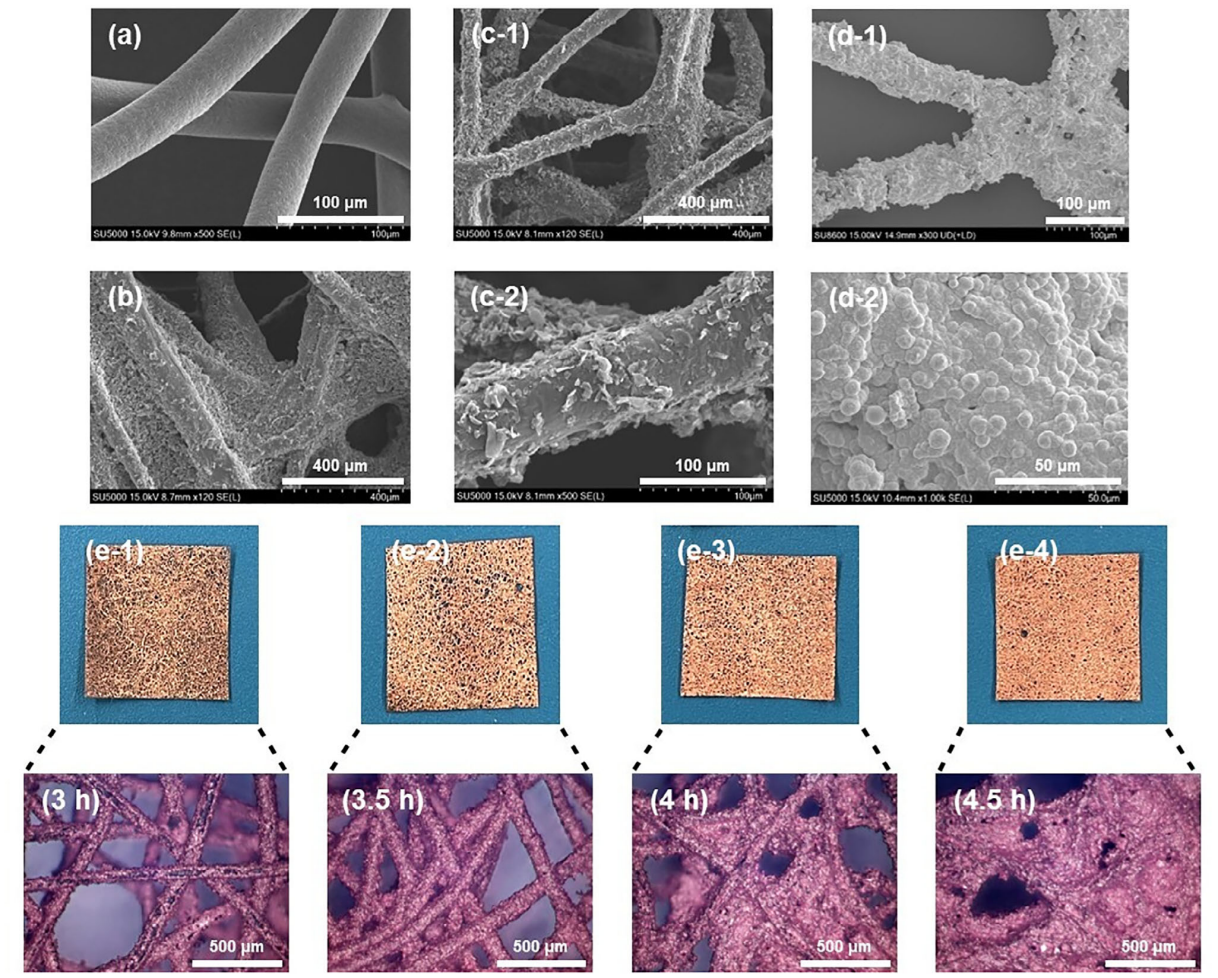
Microscopic characterization of the PGC fiber‐based pressure sensor layers. FE‐SEM images of (a) as‐spun PCL fibers, (b) PCL/graphene fibers before ultrasonication, **(c‐1, c‐2)** PCL/graphene fibers after ultrasonication, **(d‐1)** PGC fibers, and **(d‐2)** a magnified view of the electroless Cu‐plated fiber surface. Surface morphology of the fiber matrix after various plating durations: **(e‐1)** 3 h, **(e‐2)** 3.5 h, **(e‐3)** 4 h, and **(e‐4)** 4.5 h. This figure illustrates the stepwise morphological evolution of the PGC fibers throughout the fabrication process. The initial PCL fibers display smooth surfaces and high porosity, while the application of graphene introduces nanoscale roughness and enhances surface area. Post‐ultrasonication, graphene distribution becomes more uniform and conformal. Subsequent electroless Cu plating forms a metallic shell over the graphene‐coated fibers, which becomes increasingly dense and continuous with extended plating durations. These morphological transitions directly influence the sensor's conductivity, pressure responsiveness, and mechanical robustness, while preserving the overall fibrous architecture after thermal treatment.

Figure 2 provides morphological analysis of the PCL‐based fiber matrix used in the sensor layers. Figure 2a shows an optical micrograph of the as‐spun PCL mat, revealing an average fiber diameter of approximately 35 µm with surface pores formed by solvent evaporation. The overall morphology of the electrospun PCL mat is illustrated in Figure S7a, showing its uniform, porous architecture. Following multiple coating cycles, Figures 2b and S7b confirm the intercalation of graphene flakes between fibers. After thermal annealing and ultrasonic treatment in deionized water, Figure 2c‐1 demonstrates retention of the fiber network architecture, indicating that the annealing step induces localized softening for interfacial integration without collapsing the overall fibrous framework. FE‐SEM (Figure 2c‐2) verifies strong attachment of graphene flakes despite ultrasonic agitation. Elemental carbon mapping via EDS (Figure S8) further corroborates the uniform distribution of graphene on the fiber surface before and after ultrasonic cleaning.

**FIGURE 3:**
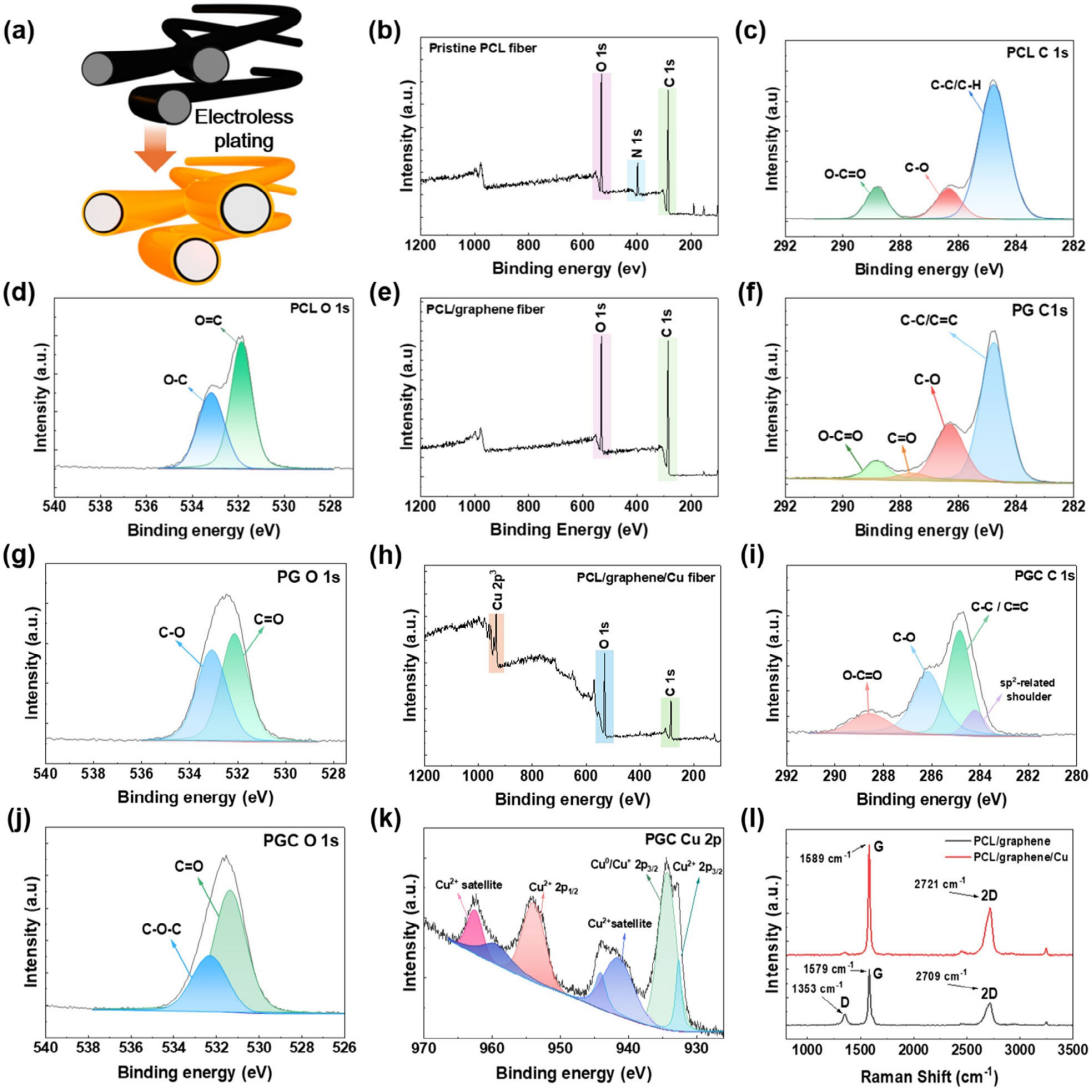
Interfacial chemical analysis and Raman characterization of PG and PGC fiber matrix. (a) schematic illustration of the graphene‐mediated electroless cu plating process. (b) XPS analysis of pristine PCL fiber. (c, d) High‐resolution C 1s and O 1s spectra of pristine PCL fiber. (e) XPS analysis of PG fiber. (f–g) High‐resolution C 1s and O 1s spectra of PG fiber. (h) XPS analysis of PGC fiber. (i–k) High resolution C 1s, O 1s, and Cu 2p secptra of PGC fiber. (l) Raman spectrum of PG and PGC fibers, showing the characteristic graphene‐related D, G, and 2D bands. XPS analysis results showed that, unlike PG, an electroless copper plating layer was formed on the surface of PGC, and changes in carbon and oxygen‐related bonding states were confirmed before and after plating. In the Raman spectrum, specific bands unique to graphene appeared, indicating that the copper layer was sufficiently thin to allow detection of the underlying graphene layer.

**FIGURE 4:**
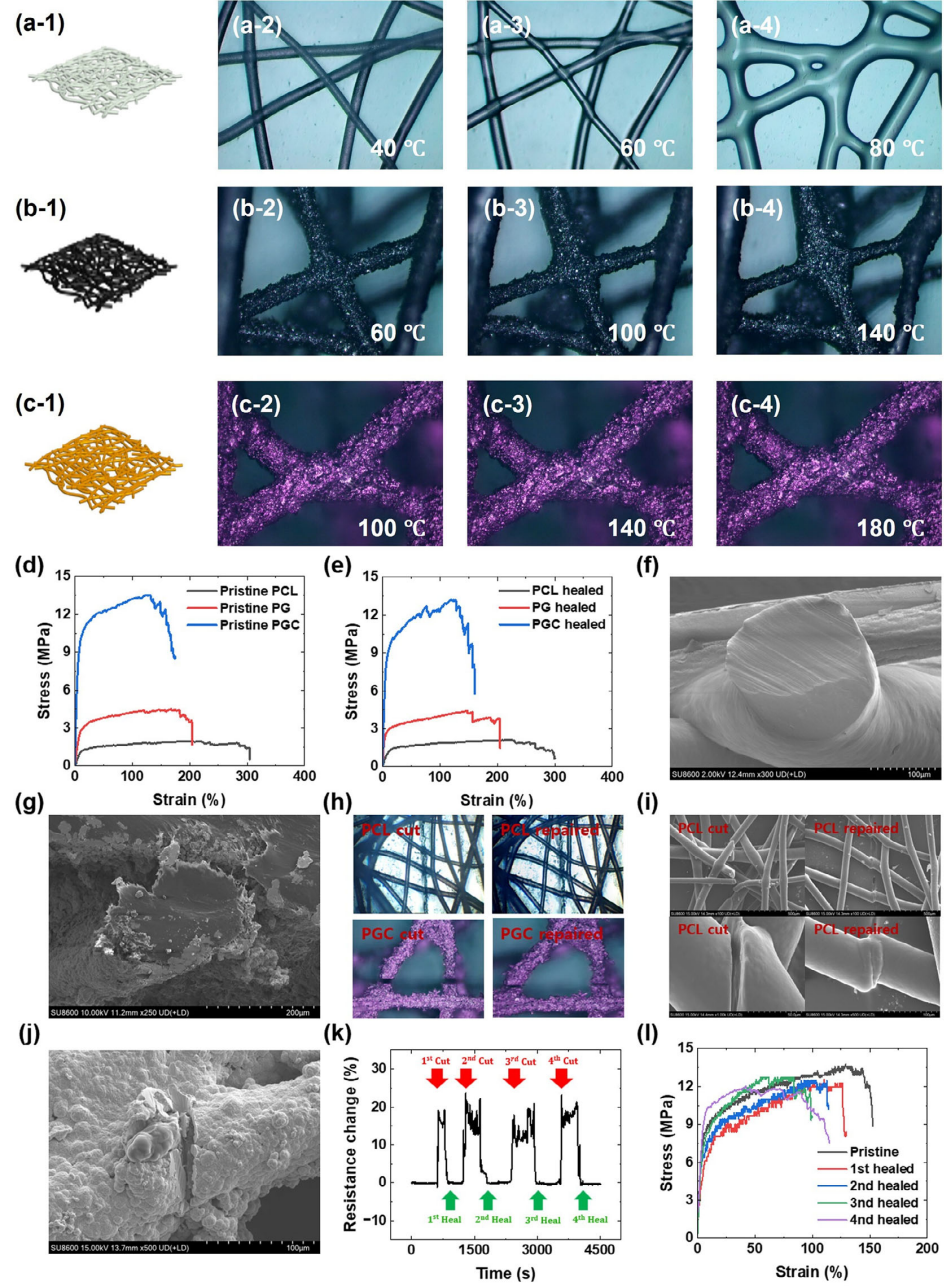
Thermally induced recovery behavior and restoration of mechanical and electrical properties in PCL, PG, and PGC fiber mats. Schematic illustrations of **(a‐1)** pristine PCL fiber sheet, **(b‐1)** graphene‐coated PCL fiber sheet, and **(c‐1)** PGC fiber sheet. Corresponding optical images of (a) the pristine PCL matrix, (b) the graphene‐coated matrix, and (c) the electroless Cu‐plated matrix. Thermal treatment of **(a‐2)** PCL fibers at 40°C, **(a‐3)** 60°C, and **(a‐4)** 80°C for 10 min; **(b‐2)** graphene‐coated PCL fibers at 60°C, **(b‐3)** 100°C, and **(b‐4)** 140°C for 10 min; **(c‐2)** PGC fibers at 100°C, **(c‐3)** 140°C, and **(c‐4)** 180°C for 10 min. (d) The stress‐strain curve of pure PCL, PG, and PGC fiber mats. (e) Stress‐strain curves of PCL, PG, and PGC fiber mats after one thermotherapy cycle. (f) A cross‐sectional SEM image of the cut PCL fiber mat. (g) A cross‐sectional SEM image of the cut PGC fiber mat. (h) Optical microscopy images of PCL and PGC fiber mats before and after thermal repair. (i) SEM image of a cut and repaired PCL fiber mat. (j) A cross‐sectional SEM image of a thermally repaired PGC fiber mat. (k) Electrical resistance variations during repeated cut‐and‐thermal‐rapair cycles. (l) Comparison of tensile strength between pristine and repaired PGC fiber matrices. This figure compares the thermally induced recovery behavior of PCL, PG, and PGC fiber mats after damage. Thermal recovery is largely dependent on the thermoplastic properties of PCL. The PGC fiber mat shows the high mechanical strength and electrical performance recovery after repeated cutting and thermal rapair cycles. Optical and SEM images confirm partial structural reconnection of the damaged area after heat treatment.

Figure S7c shows a pronounced color transition of the fibrous network from black to reddish‐brown, indicative of uniform copper deposition onto the graphene‐coated fibers. These optical changes visually demonstrate that the metal electroless plating process has been successfully completed. Figure 2d‐1,d‐2 presents FE‐SEM images of the PGC mat following electroless Cu plating, revealing the evolution from discrete Cu nanoparticle nucleation to the formation of a conformal metallic shell. The graphene interlayer plays a pivotal role in this process by acting as a catalytic mediator, enabling the electroless reduction of Cu ions without traditional Pd/Sn catalysts. Specifically, graphene's defect sites (e.g., vacancies, edges, and functional groups) and electron‐rich domains (arising from its sp^2^‐hybridized carbon lattice and pi‐electron system) facilitate the adsorption and selective reduction of Cu^2+^ ions to Cu^0^, promoting uniform nucleation and growth. This mechanism leverages graphene's high electrical conductivity and surface reactivity to lower the activation energy for Cu deposition, resulting in a homogeneous metallic shell that avoids the island‐like morphologies common in direct plating on inert polymer surfaces. EDS analyses (Figure S9) confirm homogeneous elemental distribution across the network, underscoring the effectiveness of graphene in ensuring even Cu coverage. Time‐dependent plating sequences (Figure 2e‐1–e‐4) quantitatively illustrate increasing Cu nucleation density and coalescence: initial 3 h plating yields isolated islands, whereas 3.5 h produces a continuous Cu layer enveloping each fiber while retaining inter‐fiber porosity essential for pressure sensitivity. Extended plating (4.5 h) further thickens the Cu shell without occluding the pores. Notably, the Cu coating resists removal under ultrasonic agitation, evidencing robust chemical bonding to the graphene interlayer and mechanical stability suited for repetitive pressure cycling.

FTIR spectrum of the PCL, PG, and PGC mats (Figure S10) provides insight into interfacial bonding and chemical environment changes. In the pristine PCL mat, sharp vibrations at 1735 cm^−1^ (C═O stretching), 2960 cm^−1^ (weak C─H symmetrical stretching shoulder) and 2945 cm^−1^ (medium C─H asymmetric stretching), and 1235 cm^−1^ (medium C─O─C symmetric stretching) are clearly resolved. After graphene coating (PG), the 2940 cm^−1^ CH_2_ asymmetric peak remains at the same position but shows reduced intensity, indicating graphene‐induced attenuation of PCL C─H vibrations. The 1720 cm^−1^ carbonyl peak also decreases significantly in intensity, reflecting strong interactions between graphene and PCL carbonyl groups. Similarly, the 1,235 cm^−1^ ester C─O─C peak diminishes, signifying a modified local environment due to graphene surface affinity.

Upon electroless Cu deposition (PGC), the CH_2_ asymmetry signal further decreases by 15%, demonstrating that the conformal Cu shell restricts polymer segmental motion. The carbonyl peak at 1720 cm^−1^ retains its position but shows a > 60% reduction in transmittance, consistent with strengthened interfacial interactions and possible coordination effects at the graphene–PCL interface. Additionally, the 1235 cm^−1^ ester band undergoes slight splitting and attenuates, reflecting heterogeneous dielectric environments introduced by the metallic overlayer. Together, these spectral shifts substantiate (i) graphene's modulation of PCL vibrational characteristics via noncovalent interactions and (ii) Cu plating's reinforcement of interfacial chemical coupling—mechanisms that underpin the sensor's mechanical stability and help maintain electrical pathways during thermally assisted repaired after damage, while the recovery process itself is primarily enabled by the thermally softened PCL matrix.

To determine the interfacial role of graphene in electroless copper plating, x‐ray photoelectron spectroscopy and Raman spectroscopy were performed on PG and PGC fiber mats (Figure 3). Figure 3a schematically illustrates the process of electroless copper plating following the bonding of graphene onto electrospun PCL fibers. Graphene acts as an intermediate layer formed on the fiber surface prior to plating, providing a conductive interface where copper deposition can subsequently occur. To characterize the surface chemical properties prior to graphene coating and electroless copper plating, X‐ray photoelectron spectroscopy (XPS) analysis was first performed on electrospun pure PCL fibers (Figure 3b). The full spectrum of pure PCL shows that C 1s and O 1s signals, consistent with the characteristics of polyester surfaces, appear predominantly. In the high‐resolution C 1s spectrum (Figure 3c), three major components corresponding to C─C/C─H, C─O, and O─C═O were identified. The corresponding O 1s spectrum (Figure 3d) consists mainly of C═O and C─O bond components, confirming the characteristic oxygen‐containing chemical environment of the pure PCL microfiber surface. These reference spectra serve as a reference point for changes in the interfacial chemical environment after graphene coating and electroless copper plating.

Figure 3e shows the full XPS spectrum of the PG fiber mat. C 1s and O 1s signals are predominantly observed, indicating that the graphene‐coated PCL fiber surface possesses a carbon and oxygen‐based surface composition. In the high‐resolution C 1s spectrum of PG (Figure 3f), components corresponding to C─C, C═C, C─O, C═O, and O─C═O are identified, while C─O and C═O‐related bonding components also appear in the O 1s spectrum (Figure 3g). These results demonstrate that the PG surface is not a simple carbon layer, but an interface containing both graphene‐derived graphitic carbon structures and oxygen‐containing functional groups.

In the PGC samples following electroless copper plating, copper‐related signals newly appear in the full XPS spectrum (Figure 3h). This indicates that a copper‐containing surface layer has formed on the surface of the graphene‐coated fibers. Furthermore, the high‐resolution C 1s spectrum of the PGC (Figure 3i) reveals that while carbon‐related peaks remain, sp^2^‐related shoulders are identified along with changes in some line shapes. This indicates that the graphene‐derived carbon framework remains in the interfacial region even after copper plating, and that the copper plating layer is relatively thinly formed. In the PGC O 1s spectrum of Figure 3j, components corresponding mainly to C═O and C─O─C are observed, showing that an oxygen‐containing bonding environment remains at the interface even after plating. This indicates that the oxygen‐related chemical state of the PCL/graphene‐based interface is maintained even after copper plating, and that local changes in the bonding environment occur simultaneously. The Cu 2p spectrum of the PGC (Figure 3k) more directly confirms the presence of Cu components on the surface; the accompanying satellite peaks suggest that some of the surface copper is in an oxidized state due to exposure to air. Raman analysis complements these XPS results from a structural perspective. The PG fibers exhibit D, G, and 2D bands at 1353, 1579, and 2709 cm^−^^1^, respectively (Figure 3l). This indicates the presence of a graphene‐derived graphite structure containing defects on the fiber surface. In the PGC fibers after copper plating, G and 2D bands are still clearly observed around 1589 and 2721 cm^−^^1^. In other words, this demonstrates that a thin copper plating layer is formed even after electroless plating, allowing for the detection of an intermediate graphene layer. Furthermore, the slight shift of the G and 2D bands is presumed to be due to interfacial interactions, localized strain changes, or charge transfer effects following the copper plating process.

To verify the role of the graphene interlayer, PCL/Cu (PC) and PCL/Pd‐Sn/Cu (PPC) fibers were fabricated and compared with PGC fibers (Figure S11). Surface observation via optical microscopy and SEM revealed that no Cu plating layer formed on the pure PCL surface. In the Pd/Sn‐activated PPC samples, only discontinuous Cu plating was observed, resulting in high resistance. This is presumed to be because the electrospun PCL surface is chemically inert and hydrophobic, preventing the Pd/Sn activation solution from stably and uniformly fixing to the curved microfiber surface. In contrast, the graphene‐coated PG fibers promoted Cu nucleation, enabling the formation of a more uniform Cu‐plated fiber network with lower resistance. In the tape peel test, there was almost no increase in resistance even after repeated peeling. Furthermore, SEM and EDS analysis after 100 000 load cycles revealed no interfacial delamination of the copper layer within the fiber surface, demonstrating the mechanical stability and good interfacial adhesion of the polymer/graphene/copper multilayer structure. Therefore, the graphene‐coated fiber surface provides a chemical and structural interface that is more favorable for copper deposition. In the PG samples, a defective graphitic surface containing oxygen functional groups was identified, while in the PGC samples, it was confirmed that an additional surface layer containing copper was formed while maintaining this graphene‐derived structure. Graphene acts as an interface that helps copper form in a more stable and uniform manner on the electrospun fiber matrix.

The thermal behavior of the fiber matrices directly reflects the melting characteristics of PCL (T_m_ ≈ 60°C), providing critical insights into interfacial synergy and structural resilience. Figure 4a presents (**4a‐1**) a schematic of the as‐spun PCL fiber mat, followed by FE‐SEM images at (**4a‐2**) 40°C (10 min), (**4a‐3**) 60°C (10 min), and (**4a‐4**) 80°C (10 min). At 40°C, fibers retain their as‐spun morphology; at 60°C, partial melting induces localized fusion at contact points; and at 80°C, the network fully liquefies and spreads across the substrate. Detailed optical micrographs (Figure S12) further illustrate the evolution of pore size, fiber fusion, and bulk flow as temperature increases. Graphene encapsulation significantly alters thermal dynamics. Figure 4b presents (**4b‐1**) a schematic illustration of the graphene‐coated mat, followed by FE‐SEM images at (**4b‐2**) 60°C (10 min), (**4b‐3**) 100°C (10 min), and (**4b‐4**) 140°C (10 min). At 60°C, the porous architecture remains intact; at 100°C, localized fiber expansion within the graphene shell becomes evident; and at 140°C, the PCL core expansion induces controlled deformation while the graphene overlayer maintains structural coherence. Comprehensive thermal stability imaging from 60°C to 160°C is provided in Figure S13.

The PGC mat exhibits the highest thermal endurance. Figure 4c presents (**4c‐1**) a schematic of the PGC structure, followed by SEM images at (**4c‐2**) 100°C (10 min), (**4c‐3**) 140°C (10 min), and (**4c‐4**) 180°C (10 min). Even at 180°C, the conformal graphene–Cu shell preserves the discrete fiber morphology, demonstrating exceptional high‐temperature resilience at the structural level (see also Figure S14). The conformal Cu overlayer boosts thermal diffusivity and serves as a heat sink, further stabilizing the composite structure. The sequential combination of graphene and Cu layers creates a graded thermal barrier, gradually transitioning from polymer melting to metallic rigidity and mitigating structural deformation. The high‐temperature stability of PGC fibers means that the fiber shape can be maintained by the graphene‐Cu shell upon heating. The thermally assisted recovery behavior is mainly caused by the softening of the PCL matrix inside the fiber under intentional recovery conditions. The graphene/Cu shell mainly helps to maintain interfacial alignment and conductivity continuity. To quantify the impact on electrical functionality, real‐time resistance was measured during thermal cycling to 200°C (Figure S15). The PGC mat exhibits a gradual, mild increase in resistance up to ∼150°C, reflecting slight thermal expansion of the PCL core and minor perturbations in the Cu shell conducting pathways. Beyond this threshold, resistance rises sharply between 150°C and 200°C, attributable to enhanced polymer expansion exerting strain on the metallic shell and transient disruption of electron percolation networks. Upon cooling from 200°C to room temperature, the resistance decreases along a distinct hysteresis path—likely due to differential contraction rates of the polymer and metal layers—but notably, returns to its initial baseline value at 0°C. This reversible electrical behavior underscores the mat's exceptional thermal tolerance in terms of reversible electrical response during heating and cooling and mechanical integrity, as the graded PGC architecture effectively accommodates thermal stresses without permanent loss of conductivity. Such resilience is critical for ensuring sensor reliability under fluctuating temperature environments.

Mechanical assessments reveal the synergistic reinforcement imparted by graphene and Cu coatings. Tensile testing (Figure 4d) shows that the pristine PCL fiber mat exhibits an ultimate tensile strength (UTS) of 2.2 MPa with a fracture strain of approximately 320%, characteristic of a highly ductile polymer. After graphene encapsulation (PG), the UTS increases to 4.9 MPa while the fracture strain decreases to ∼210%, reflecting enhanced load transfer via graphene–PCL interfacial interactions and moderated polymer deformation. Subsequent electroless Cu deposition (PGC) further elevates the UTS to 13.7 MPa, accompanied by a reduced fracture strain of ∼150%, indicative of a continuous metallic scaffold bearing the majority of the mechanical load at the expense of extensibility. These quantitative improvements confirm that the hierarchical PGC architecture dramatically enhances mechanical strength while maintaining sufficient ductility for reliable sensor operation under stress. Mechanistically, the incorporation of graphene flakes at fiber interfaces increases interfacial shear strength through improved load transfer, as predicted by micromechanical composite models. The subsequent Cu shell contributes additional reinforcement by forming a continuous metallic network that bridges adjacent fibers, effectively distributing tensile loads and inhibiting crack initiation and propagation. Fracture analysis indicates that the graded stiffness between the polymer core and metallic shell mitigates stress concentration, delaying the onset of brittle failure. Moreover, the retained elongation (∼150% at UTS) in PGC mats suggests that the polymer–metal interface remains cohesive under large strains, enabling crack‐closure and helping preserve conductive pathways during thermally assisted recovery of electrical conductivity and sensing performance after damage. The recovery process itself is primarily driven by thermoplastic softening and reflow of the PCL matrix rather than by graphene or Cu. These multiscale reinforcement mechanisms—interfacial bridging, metallic scaffolding, and graded stiffness—collectively underpin the sensor's ability to tolerate mechanical deformation cycles without performance degradation, a critical requirement for wearable and dynamic pressure‐sensing applications.

After one damage and thermal repair cycle, all three samples showed tensile strength similar to pristine (Figure 4e), confirming that the recovery process is fundamentally enabled by the thermoplastic properties of PCL. The repaired PGC mat, however, maintained substantially higher stress levels than the repaired PCL and PG mats. This means that the graphene/Cu shell structure helps maintaining the fiber matrix structure after heat treatment. OM and SEM analysis were performed to visually show the thermoplastic recovery properties of PCL and the functional recovery of PGC. The PGC sample shows the highest UTS recovery rate and elongation recovery rate (Figure S16). This means that the fiber structure into which graphene/Cu is introduced maintains excellent mechanical integrity even after recovery. Thermal rejoining after damage is fundamentally driven by the thermoplastic properties of PCL, whereas the graphene/Cu layer contributes to maintaining mechanical strength and stability after repair. The cross‐sectional SEM image (Figure 4f) of the cut PCL shows the cleanly broken polymer cross section. The cut PGC cross section retains the core–shell structure of the coated graphene and plated Cu layer structure at the damaged interface (Figure 4g). Optical microscope images of the cut and repaired area (Figure 4h) show the reconnection of the damaged PCL and PGC fibers through heat treatment. However, it can be seen that the local area damaged by the blade is not completely healed. High‐magnification SEM images of PCL samples before and after restoration (Figure 4i) show recombination of broken polymer surfaces after heating. These results show that the thermal recovery is driven by the softening of the PCL matrix, while the graphene/Cu layer helps to maintain the structural continuity and mechanical function of the repaired fibers. In the recovered PGC sample, the damaged area remains partially and is not fully restored to the original fully plated layer structure (Figure 4j). The opposing fracture surfaces are brought back into close contact after heat treatment because of softening and local reflow of the PCL matrix inside the PGC. This means that the recombination is not caused by the melting of graphene or Cu layers themselves but by heat‐induced recombination into the PCL skeleton, and renewed local contact between existing conductive shells. It can be visually confirmed that the cut PGC fiber matrix was thermally rejoined at 80°C (Figure S17). To confirm the electrical recovery, the resistance of the same PGC specimen was measured during four cutting and recovery cycles (Figure 4k). Each cutting showed a resistance increase of 20%–25%, indicating that the redundant conduction path was broken. After recovery, the resistance recovered to within ±5% of the initial value, showing that a continuous electron path is restored. Diels‐Alder reactions that require conventional heating or catalyst may degrade in performance during damage‐recovery, and require slow and reversible chemical reactions [28, 29]. On the other hand, the damaged PGC was physically and electrically restored within 15 min. To evaluate the mechanical recovery capacity of PGC fibers, tensile tests were performed on intact and thermally restored mats (Figure 4l). Despite up to four consecutive damage‐recombinations at the same site, the tensile strength of the recovered PGC mat remained above 90% of its original value. The fracture strain was reduced by only about 10% per cycle, presumably due to the insignificant loss of Cu nanoparticles at the fracture interface. PCL melting enables interdiffusion and re‐entanglementation of polymer chains, resulting in repetitive functional repair without relying on specific chemical functions that can become tired or hydrolyzed over time [30]. The PGC fiber matrix shows a repeatable electrical and mechanical restoration of electrical conductivity after damage. Under high‐temperature exposure conditions, graphene‐Cu shells help preserve the entire fiber morphology and conductive skeleton of the PGC structure. By utilizing the low melting point of PCL within a hierarchical graphene‐copper framework, this sensor can recover electrical resistance and mechanically damaged areas even after damage at 80°C. However, since the severed area is not completely geometrically restored, it is promising for repairable and reusable sensing applications where thermal recovery by external stimuli is permitted.

**FIGURE 5:**
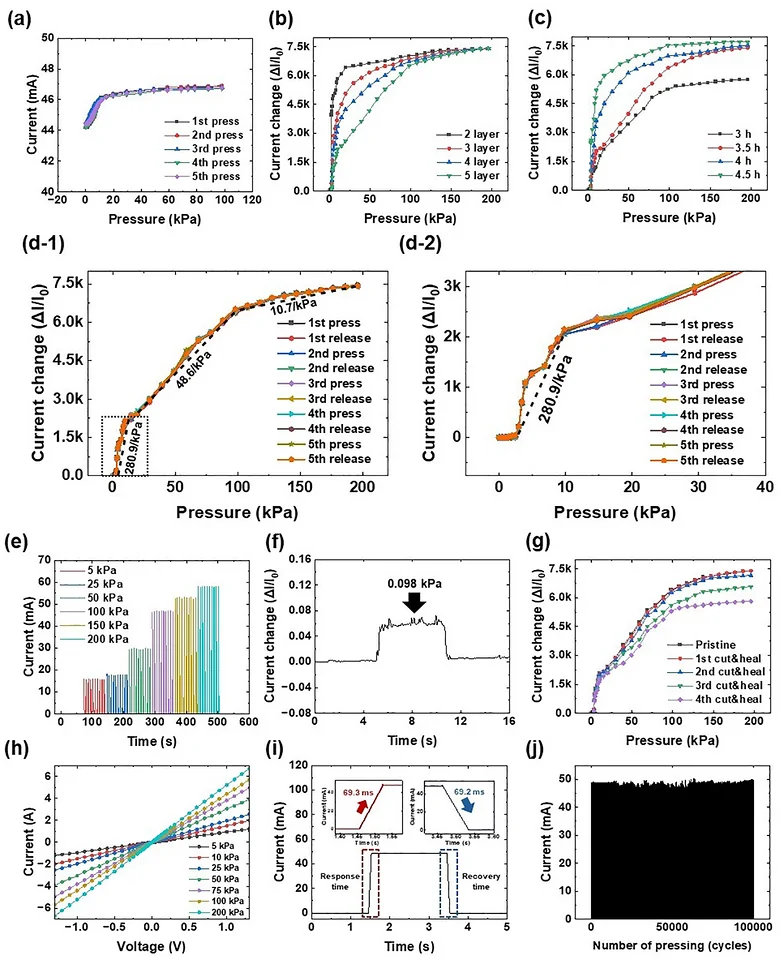
Sensing performance of the PGC fiber matrix sensor: (a) Current response of a single‐layer sensor under incremental pressures from 0.098 to 100 kPa. (b) Influence of the number of PGC layers on current output. (c) Effect of copper plating time on sensor response. **(d‐1)** Current output from a five‐layer sensor under five successive press–release cycles. **(d‐2)** Magnified plot showing current variation in the low‐pressure region (0.098–40 kPa). (e) Cyclic response of a five‐layer sensor under varying pressures from 5 to 200 kPa. (f) Response to the minimum detectable pressure of 0.098 kPa. (g) Current variation at a fixed site through repeated thermal repair cycles. (h) *I–V* characteristics under pressures of 5, 25, 50, 75, 100, and 200 kPa. (i) Response and recovery times for a 100 kPa load. (j) Long‐term durability of the five‐layer sensor over 100 000 loading cycles at 100 kPa. This figure illustrates the sensor's high sensitivity, broad detection range, repeatability, fast response, and exceptional cycling endurance, validating its suitability for practical pressure sensing applications.

The electromechanical response of the PGC fiber matrix sensor was evaluated across multiple configurations and conditions (Figure 5). In a single‐layer sensor (Figure 5a), the continuous conductive network yields a high baseline current even in the absence of pressure. When subjected to pressures ranging from 0.098 to 100 kPa (5 cycles each), the single‐layer device exhibits minimal current change, with sensitivity values of 0.033 kPa^−1^ in the 0–10 kPa regime and 2.3 × 10^−4^ kPa^−1^ in the 10–100 kPa regime. The limited contact area between fibers in this configuration constrains its sensitivity. To enhance sensitivity, multilayer sensors composed of 2–5 stacked PGC mats were tested (Figure 5b) [31]. Unlike the single‐layer device, multilayer configurations show pronounced current modulation upon pressing. At zero pressure, the layers remain electrically isolated, and only a low leakage current flows via the AgNW‐coated PCL support. As pressure is applied, successive layers make contact, resulting in abrupt increases in current. Increasing the number of layers augments the effective contact area between PGC mats, improving linearity and sensitivity [32].

To provide a quantitative interpretation of the proposed layer‐by‐layer contact mechanism, the compression behavior of 2‐, 3‐, 4‐, and 5‐layer PGC sensors was further analyzed (Figure S18). As the number of stacked layers increased, the pressure‐sensing range gradually broadened. The pressure‐dependent thickness change and corresponding compressive strain varied systematically with layer number, reflecting differences in structural densification behavior under compression. The 2‐layer sensor compressed and saturated more rapidly at low pressure, whereas the 5‐layer sensor exhibited more gradual compression over a wider pressure range. These results suggest that multilayer sensors with more interlayer interfaces and larger compressible gap volume can form conductive junctions sequentially before full compaction is reached, thereby delaying signal saturation and expanding the pressure‐sensing range. The influence of electroless plating time on sensor performance was also investigated using five‐layer devices (Figure 5c). Devices plated for 3 and 3.5 h achieve linearity coefficients (R^2^) of 0.885 and 0.905, respectively, while 4 and 4.5 h plating yield lower R^2^ values of 0.7635 and 0.65 due to pore occlusion and reduced inter‐fiber contact. Although the 4 and 4.5 h devices demonstrate higher sensitivity at low pressures, their nonlinearity compromises measurement accuracy. Therefore, a 3.5 h plating time was identified as optimal for balancing sensitivity (281 kPa^−1^ in 0–10 kPa, 49 kPa^−1^ in 10–100 kPa, and 11 kPa^−1^ in 100–200 kPa) and linearity over a broad pressure range (0.098–200 kPa) (Figure 5d). Reproducibility tests were conducted by applying cyclic pressures from 5 to 200 kPa (Figure 5e). The sensor maintains consistent current responses under repeated loading, confirming reliable performance [33, 34]. Further, a fixed pressure of 60 kPa was applied repeatedly at 0.01 V bias using a pushing tester (Figure S19); the recorded current remained stable, demonstrating high measurement fidelity. The minimum detectable pressure is as low as 0.098 kPa (1 g weight), with a measurable current change observed at this threshold (Figure 5f).

The effect of thermally assisted functional recovery on sensing performance was assessed by comparing devices before and after up to four cut‐and‐thermal‐repair cycles at the same location (Figure 5g). After one or two repair cycles, current–pressure characteristics closely match the pristine sensor. After three or four cycles, slight decreases in current response are observed, attributable to partial loss of Cu coating at the cut interface, yet the sensor retains measurable pressure sensitivity. Current–Voltage (*I–V*) measurements under various pressures (5, 25, 50, 75, 100, 200 kPa) reveal a linear relationship across the tested voltage range (Figure 5h), revealing predictable sensor behavior and ease of calibration. Dynamic response tests at 100 kPa yield a response time of 69.3 ms and a recovery time of 69.2 ms (Figure 5i), comparable to human muscle reflex times (60–70 ms), underscoring the sensor's rapid reaction capability [35, 36]. Long‐term stability was verified by subjecting a five‐layer sensor to 100 000 cycles of 100 kPa loading (Figure 5j). The current remained at 48 mA with negligible drift, validating exceptional mechanical and electrical durability for extended operation. To assess thermal durability under cyclic loading, sensors fabricated from PGC mats thermally treated at 25°C, 60°C, 100°C, 140°C, and 180°C were tested at 100 kPa (Figure S20). All samples exhibited stable current outputs, with the 180°C‐treated sensor showing only a 2.6% reduction relative to the pristine device, confirming robust sensing performance after high‐temperature exposure. Additionally, long‐duration hold tests at various pressures (5, 25, 50, 100, 200 kPa) for 10 s, repeated five times, showed virtually identical current levels for each pressure step (Figure S21), reaffirming the sensor's outstanding reproducibility under static loading. The Video S1 also provides compelling in situ evidence of the developed sensor's mechanical stability and its uniform sensing characteristics. The consistent signal output observed during repeated pressure applications clearly illustrates the device's structural integrity and reliable performance, while the thermally assisted repair itself is primarily enabled by the PCL matrix and the graphene/Cu shell mainly helps preserve conductive continuity after damage. The multilayer architecture enhances sensitivity through a sequential contact mechanism [37, 38]: as external pressure increases, discrete PGC mat layers progressively engage, creating new conductive junctions and amplifying current change. The layered configuration effectively converts macroscopic pressure into localized microcontact events, where the density of conductive pathways scales with applied force. Electroless plating time influences pore size and conductive shell thickness, which in turn modulate sensor performance. Shorter plating (3 h) yields a thinner Cu shell that preserves high porosity, maximizing fiber–fiber contact sensitivity at low pressures. Extended plating (> 4 h) diminishes porosity and increases shell stiffness, raising the threshold pressure for contact formation and reducing dynamic range. Thus, the optimal 3.5 h plating balances shell continuity for reliable percolation with sufficient pore volume for pressure‐induced contact modulation. The sensor's ultralow detection limit (0.098 kPa) arises from the high gauge factor inherent to the PGC network: minor pressure increments induce measurable changes in tunneling distance and contact resistance across graphene–Cu junctions. The rapid response (≈70 ms) may reflect minimal viscoelastic damping in the PCL core and efficient charge transport within the metallic shell. Reproducibility under repeated loading underscores the structural recovery of the multilayer contact network during unloading and the mechanical plasticity of the metallic overlayer. The AgNW electrodes maintain stable interface conduction, while the graphene–Cu network is proposed to accommodate repeated deformation via reversible slip and recontact at nanoscale junctions.

Following our demonstration of the PGC sensor's exceptional electromechanical performance, we compared its key pressure sensor metrics against representative piezoresistive pressure sensors from the literature (Table 1) [37, 38, 39, 40, 41, 42]. While certain reported devices achieve extremely high sensitivities—such as AgNW/polybutadiene‐urethane(PBU) composites at 888.8 kPa^−1^ [39] or carbon‐nanotube‐based architectures at 571.6 kPa^−1^ [40]—these systems are typically limited to narrow pressure ranges (< 100 kPa) and often omit critical performance parameters such as minimum detection limit, high‐temperature stability, long‐term cycling reliability, mechanical strength, or damage recovery capability. In contrast, our PGC microfiber sensor simultaneously delivers a sensitivity of 280.9 kPa^−^^1^ across an exceptionally broad detection window (0.098–200 kPa) and a very low detection threshold of 0.098 kPa. Its 69 ms response time matches or exceeds most prior reports, yet it also uniquely combines rapid, moderate‐temperature thermally assisted functional recovery after damage, high‐temperature structural and electrical stability under heating (up to 180°C), mechanical reinforcement (UTS of 13.7 MPa), and stable performance over 100 000 loading cycles. The thermally assisted recovery is primarily driven by the PCL matrix and the graphene/Cu shell mainly supports conductive and structural restoration after damage. To evaluate the signal stability of the PGC pressure sensor, a constant load application test was additionally performed (Figure S22). A constant pressure was applied to the sensor for approximately 8000 s, and the current signal was monitored in real time. The sensor demonstrated stable operation even under prolonged constant pressure conditions by maintaining a stable current without abrupt signal fluctuations. Normalized drift was calculated by determining the change between the initial and final current values under each load condition, and the drift rate was obtained by analyzing the holding time of the normalized drift. The sensor's drift rate decreased from 1.94 to 0.054%h^−^^1^ as the applied load increased. This indicates that the conductive contact network stabilized when sufficient interlayer contact was achieved under high loads. This demonstrates that the sensor possesses good signal stability even under prolonged load application. Electrical and mechanical stability when bending is applied to the PGC sensor was examined (Figure S23). The developed sensor showed almost the same current change value as the flat state when a load was applied at a certain bending radius. It was confirmed that when 50 kPa was repeatedly applied under all bending conditions (10, 5, 3 mm), the initial current change value recovered stably. This means that the sensor's pressure sensing ability is maintained even with a certain bending. Slight noise occurred during the repeated bending cycle of about 10 mm, but the peak response retention rate remained almost uniform. In addition, a shear shift test was conducted by moving the weight little by little with a constant load applied with the metal weight (Figure S24). It maintained stable current change output during repeated cycles with shear shifts of up to 3 mm at 50 kPa. These results show that the conductive fiber network is electrical and mechanically stable even under shear forces.

**TABLE 1:** Performance comparison with the recently reported piezoresistive pressure sensors.

Feature	Sensitivity (kPa^−1^)	Pressure range (kPa)	Limit of detection (kPa)	Response time (ms)	References
Semi‐cylinder AgNW/PBU	888.79	< 100	0.004608	66	[39]
3D CNT structures	571.64	10–50	N/A	85	[40]
LiCl‐filter paper (FP), CNTs‐FP layers	0.73	0.1–100	0.1	562	[41]
Gadient slant structure	N/A	0.003–100	0.003	45	[42]
PVA/SA electrospun nanofiber	0.54	< 300	0.0022	28.7	[43]
Graphene/silicone rubber	34.15	0.036–2.79	0.036	34	[44]
PGC fiber matrix layers	280.9	0.098–200	0.098	69	This work

To evaluate the reproducibility of the fabricated fiber‐based multilayer pressure sensor, the initial resistance and current change with pressure of independently fabricated PGC sensors were measured (Figure S25). The histograms of the initial resistance values of the 57 sensors showed a relatively narrow distribution, and the average initial resistance was 584 Ω, indicating that the resistance difference between the devices was small. In addition, the five independently fabricated sensors exhibited similar current change values across the entire pressure range. This demonstrates the reproducibility of the PGC pressure sensor in both initial resistance and pressure sensing characteristics, despite the random structure of the electrospun fiber network. These extensive functionalities highlight the PGC sensor's well‐harmonized, multifunctional configuration, demonstrating that an expanded detection range is achieved without compromising sensitivity and that structural endurance is preserved alongside rapid response times.

**FIGURE 6:**
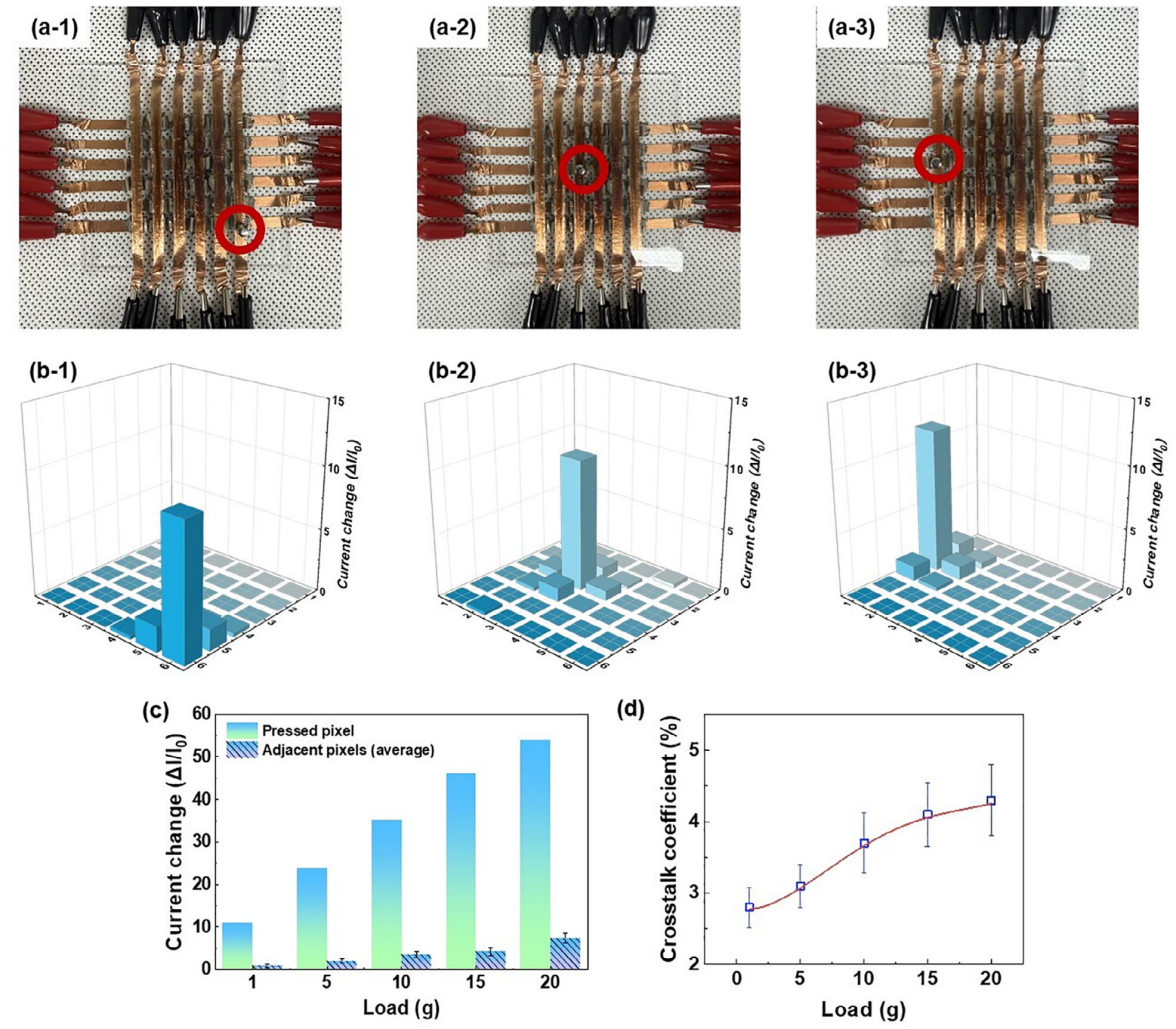
Single‐pixel load test and crosstalk analysis of a 6 × 6 PGC sensor array. **(a‐1∼a‐3)** Images showing a 1 g weight placed on a single pixel in a 6 × 6 PGC sensor array, with the pressed pixel indicated by a red circle. **(b‐1∼b‐3)** 3D map of current change when a load is applied to a single pixel within the array. (c) Comparison of the normalized current change of the pressed pixel and the average response of adjacent pixels under applied loads of 1, 5, 10, 15, and 20 g. (d) Crosstalk coefficient according to load, defined as the ratio of the average response of adjacent pixels to the response of the pressed pixel. Since the PGC pressure sensor array maintains local pressure detection while minimizing signal leakage to adjacent pixels, it is suitable for spatially resolved pressure mapping.

To verify the 6 × 6 PGC sensor array, tests were performed by applying loads to single pixels within the array, and crosstalk between pixels was quantitatively analyzed (Figure 6). As shown in the optical images (Figure 6a‐1–3), local loads were applied to pixels at representative positions of the array, including corner, edge, and center pixels. The 3D current maps (Figure 6b‐1–3) showed that normalized current changes were consistently concentrated on the pressed pixels, while surrounding pixels exhibited weak current changes. This demonstrates that the array transmits pressure input locally to the target detection device without severe signal diffusion to adjacent sensors. Various low loads of less than 20 g were applied to single pixels within the array, and the current change values and the average values of adjacent pixels were quantitatively analyzed (Figure 6c) The directly pressed pixels exhibited significantly larger current changes than the adjacent pixels, and their responses increased gradually with increasing load. On the other hand, the current changes in adjacent pixels were relatively small, confirming that most current changes occurred in the pixels directly subjected to the load. The crosstalk coefficient was calculated as the ratio of the average current change of adjacent 8 pixels to the current change of the compressed pixel (Figure 6d). Error bars represent the standard deviation of 10 repeated measurements. As the applied load increased, the crosstalk coefficient increased slightly but remained at a low level in the range of approximately 2.8%–4.3%. This shows that while mechanical and electrical coupling with adjacent pixels may increase slightly as the external load intensifies, the dominant response is still maintained at the compressed pixel. This demonstrates that interference between pixels is small upon load application, proving that the PGC array is suitable for accurate spatial pressure mapping of local loads.

**FIGURE 7:**
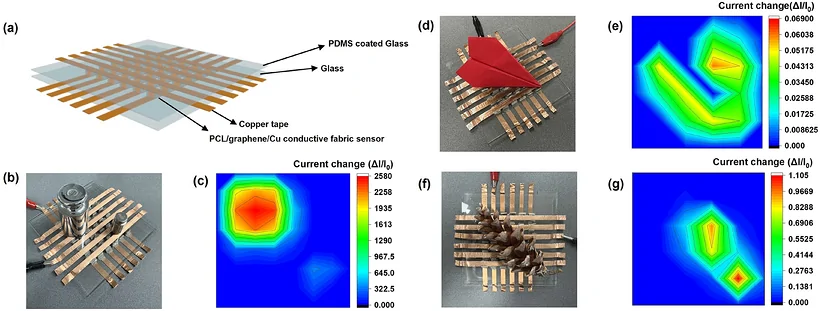
Evaluation of pressure sensor array performance using various objects: (a) Schematic diagram of the fabricated multi‐pixel sensor array. (b) Sensor array under applied weights of 200 and 20 g. (c) Corresponding current distribution heatmap for panel (b). (d) Sensor array with a paper plane (1.46 g) placed on top. (e) Heatmap showing the spatial current response to the paper plane. (f) Sensor array with a pinecone (4.67 g). (g) Resulting current distribution heatmap for panel (f), confirming detection of light, irregularly shaped objects. These measurements confirm that the array distinguishes weight and shape differences across its pixels, demonstrating high spatial resolution, low detection threshold, and applicability for diverse pressure‐mapping scenarios.

To demonstrate system‐level integration, we constructed a 6 × 6 PGC sensor array on a 100 × 100 mm glass substrate. Bottom electrodes were patterned using fixed copper tape traces, while a matching copper grid was affixed to a 100 µm‐thick PDMS‐coated glass top plate to serve as the counter electrode and to distribute load more locally (Figures 7a and S26). The PDMS layer minimizes pressure spread across the array, preventing spurious activation of non‐loaded pixels and improving spatial resolution [45]. Various objects—including pinecones (4.67 g), steel weights (10 and 200 g), and custom‐folded paper planes (1.46 g)—were sequentially placed on the array (Figure S27). Each object induces a distinct pressure profile that activates only the underlying pixels. For the 200 g weight, a broad region of high current change is observed (Figure 7b,c), whereas the 10 g weight yields a smaller, well‐defined footprint. The lightweight paper plane and pinecone generate highly localized responses at their contact points (Figures 7d–g, S28 and S29), demonstrating the array's ability to detect low‐mass objects with precise positional accuracy. Quantitative mapping shows that loaded pixels exhibit current increases up to 150%, while adjacent non‐loaded pixels remain at baseline within ±2% of the zero‐pressure current under 0.01 V bias. The minimal crosstalk and steep current gradients confirm superior pixel independence. These demonstrations confirm that the PGC sensor can be readily scaled into high‐density arrays capable of precise, low‐noise pressure mapping across irregular surfaces [46].

To demonstrate the practical applicability of the PGC sensor array, we performed a self‐powered switching proof‐of‐concept test by integrating a triboelectric nanogenerator (TENG), a bridge rectifier, and an LED circuit (Figure S30, Video S2). When pressure was applied to the PGC sensor array, the device acted as a pressure‐sensing switch to turn on the LED. This result confirms that the fabricated PGC sensor array can be directly connected to external electronic components and has the potential to be utilized in self‐powered interactive sensing applications. Such capabilities open avenues for applications in robotic tactile skins, where conformable arrays must detect nuanced contact patterns; interactive touch panels with pressure‐sensitive control; and structural health monitoring in industrial settings, where distributed force sensing can preemptively flag overloads or defects [47, 48]. Collectively, the 6 × 6 array exemplifies the platform's versatility and readiness for integration into multifunctional sensing systems.

## 3 Conclusion

This study demonstrates that a carefully engineered PGC microfiber network can overcome longstanding tradeoffs in pressure sensor design, achieving simultaneously ultrahigh sensitivity (> 280 kPa^−1^), an exceptionally broad detection range (0.098–200 kPa, R^2^ = 0.905), rapid electromechanical response, long‐term durability over 100 000 cycles at 100 kPa, and stable operation with preserved structural and electrical functionality after heating to 180°C. Central to this performance is the atmospheric plasma‐activated graphene interlayer, which seeds uniform, conformal electroless Cu shells that establish robust electrical percolation pathways and reinforce fiber interfaces (UTS up to 13.7 MPa) while retaining the intrinsic porosity required for pressure‐dependent contact modulation. This graphene‐mediated electroless deposition process provides a Pd/Sn‐free approach for achieving more homogeneous Cu nucleation on electrospun polymer fibers under mild conditions, and may help reduce the non‐uniformity and pretreatment complexity associated with conventional Pd/Sn activation. Moreover, the low melting point of PCL uniquely enables a straightforward thermally assisted repair mechanism at 80°C, allowing recovery of over 95% of electrical conductivity and over 90% of mechanical strength within 15 min, although complete geometric closure of the cut region is not achieved. While thermal recovery is intentionally activated by melting of PCL core, high temperature stability of PGC system means that the entire fiber morphology and conductive skeleton are maintained by graphene‐Cu shell during heating. Therefore, graphene/Cu layer should be understood as electrical and structural stable layer, not primary recovering medium. By optimizing Cu plating duration (3.5 h), we balanced shell continuity with pore volume to maximize both linearity and sensitivity across multiple pressure regimes. System‐level integration of these PGC mats into a 6 × 6 array confirmed precise spatial mapping of minute loads (down to 1 g) with minimal crosstalk and 16 pixels/cm^2^ resolution, underscoring the platform's scalability and potential for practical integration. These findings highlight the power of hierarchical structuring, mild surface activation, and graphene‐mediated interfacial engineering to reconcile formerly conflicting sensor requirements. The resulting PGC platform offers a versatile blueprint for next‐generation repairable and reusable wearable interfaces, flexible pressure‐sensing platforms, robotic tactile skins, and distributed force‐sensing networks in industrial settings—where high‐density, reliable, and multifunctional pressure detection is paramount. Future work could explore integration with sustainable or recyclable polymer platforms and scaling to large‐area production for cost‐effective commercialization, further expanding the impact of this innovative materials strategy.

## 4 Experimental Section

### 4.1 Materials and Reagent

PCL pellets were purchased from Sigma–Aldrich. Dichloromethane was obtained from Daejung Chemicals & Metals Co., Ltd. Conductive graphene dispersion (23 wt.%) was sourced from Graphene Supermarket. To prepare a 3 wt.% coating solution, the graphene dispersion was diluted in n‐butyl acetate (Sigma–Aldrich). For electroless copper plating, Cupro‐Build solution was obtained from Maru Enhanced Materials, while sodium hydroxide (NaOH, assay 98%) and formalin (35% assay) were acquired from OCI Company Ltd. AgNWs dispersed in isopropyl alcohol (IPA) were provided by C3 Nano Inc. (USA). All reagents were used without further purification. All experiments related to material preparation were performed in a standard fume hood.

### 4.2 Fabrication of Conductive Fiber Matrix with Thermally Assisted Functional Recovery

6 g of PCL pellets were dissolved in 23 mL of dichloromethane under stirring at 60°C and 90 rpm for 6 h. Once a homogenous solution was achieved, the solution was loaded into a 20 mL syringe fitted with a 17‐gauge metal nozzle (1.23 mm diameter). Electrospinning was performed using a high‐voltage electrospinning system (ESR200R2, NanoNC, Korea) with an applied voltage of 8 kV, a feed rate of 0.8 mL/h, and a 150 mm nozzle‐to‐collector distance. The resulting PCL fiber mat was dried in a vacuum oven at 40°C for 30 min. The dried mat was carefully peeled from the aluminum foil using tweezers. Prior to graphene coating, the PCL fiber mat underwent atmospheric pressure plasma treatment using a gas mixture of argon (Ar) and oxygen (O_2_) to introduce polar functional groups such as ─OH and ─COOH onto the fiber surface, thereby enhancing graphene adhesion. Plasma treatment was conducted at room temperature under a flow of Ar/O_2_ (95:5 vol%) for 60 s using a plasma generator, ensuring surface activation without compromising fiber morphology. Following this activation step, the mat was dip‐coated in the 3 wt.% graphene dispersion. This process was repeated until a uniform graphene coating was achieved across the fiber surface. The coated mat was dried at 60°C in a convection oven. Ultrasonic treatment in deionized water was then applied to remove loosely bound graphene flakes. Finally, the mat was dried at 40°C for 30 min.

To promote catalytic activity, uniform copper deposition, and strong interfacial adhesion, the graphene‐coated PCL mat relied on the intrinsic catalytic properties of the uniformly distributed graphene layer. The presence of polar functional groups introduced via atmospheric pressure plasma treatment, combined with the high surface conductivity of graphene, facilitated spontaneous nucleation and conformal copper film growth without the need for additional chemical activation steps. For electroless plating, 108 mL of deionized water, 12 mL of Cupro‐Build, 1.08 mL of formalin, and 0.96 mL of NaOH were mixed and stirred at 45°C. The graphene‐coated PCL mat was immersed in the plating solution for 3, 3.5, 4, or 4.5 h. After plating, the mat was rinsed with deionized water, dried at 40°C for 10 min, and cut into 10 × 10 mm patches.

### 4.3 Fabrication of Multilayer Pressure Sensor

A PCL solution was prepared by dissolving 4 g of PCL pellets in 21 mL of dichloromethane under stirring at 60°C for 2 h. A 2 g aliquot of the solution was spin–coated onto a 25 × 25 mm glass sheet at 1500 rpm to create a thin and flat substrate. The film was dried at 60°C for 5 min and immediately scratched to introduce linear defects before being cured at room temperature for 10 min. The cured PCL film was folded into a zigzag configuration. The previously prepared PCL solution was applied to the contact areas between the PCL support and the PGC fiber matrix to construct a five‐layer sensor structure. Finally, to impart conductivity to the PCL support, a 0.3 wt.% AgNW solution was spray‐coated uniformly and dried at room temperature for 10 min.

## Author Contributions

**ByungJun Kim**: methodology, data curation, formal analysis, investigation, writing – original draft. **Su Bin Choi**: conceptualization, methodology. **Yeonwook Jeong**: investigation, validation. **Jeong‐Won Yoon**: project administration. **Seung‐Boo Jung**: project administration. **Jong‐Woong Kim**: writing – review and editing, conceptualization, data curation, supervision, project administration, funding acquisition.

## Conflicts of Interest

The authors declare no conflicts of interest.

## Supporting information




**Supporting File 1**: advs77794‐sup‐0001‐SuppMat.docx.

**Supporting File 2**: advs77794‐sup‐0003‐SuppMat.docx.

**Supporting File 3**: advs77794‐sup‐0003‐VideoS1.mp4.

**Supporting File 4**: advs77794‐sup‐0004‐VideoS2.mp4.

## Data Availability

The data that support the findings of this study are available from the corresponding author upon reasonable request.
