# Supplementary material for: High‐Performance Piezoresistive Sensors via Pd/Sn‐Free Graphene‐Catalyzed Metallization of Thermally Repairable Microfiber Scaffolds
Source: Adv Sci (Weinh). 2026 Sep 16:e77794. Online ahead of print. doi: 10.1002/advs.77794 (PMC13583221; doi:10.1002/advs.77794)
Supplement: Supplementary file 1 [file ADVS-9999-e77794-s002.docx]

Copyright WILEY-VCH Verlag GmbH & Co. KGaA, 69469 Weinheim, Germany, 2013.

Figure of merit

High-Performance Piezoresistive Sensors via Pd/Sn-Free Graphene-Catalyzed Metallization of Thermally Repairable Microfiber Scaffolds

*ByungJun Kim, Su Bin Choi, Yeonwook Jeong, Jeong-Won Yoon,* Seung-Boo Jung, Jong-Woong Kim**

To comprehensively evaluate the pressure sensing performance of the developed PGC sensor, the performance of recently reported pressure sensors was compared using two graphs. First, we compared sensitivity and linear detection range, because these two parameters are key factors that determine a pressure sensor’s ability to detect minute pressure changes while maintaining stable detection over a wide operating range. In addition, response time and repeat durability were evaluated to verify the sensor’s dynamic detection capability and long-term operational reliability. Through this, the detection performance of the PGC sensor was evaluated in terms of sensitivity, operating range, response speed, and durability.

**Figure 1.** Comparison of sensitivity and linear sensing range of the developed PGC pressure sensor with previously reported pressure sensors.

To quantitatively compare and evaluate the sensitivity of the developed PGC sensor, we compared its sensitivity and linear detection range with those of representative pressure sensors reported in recent literature (**Figure 1**). Since pressure sensors generally vary in sensitivity depending on pressure, instead of using only the maximum sensitivity, we separately represented the sensitivity corresponding to each linear pressure range. The PGC sensor demonstrates a high sensitivity of 280.9 kPa⁻¹ in the low-pressure region (0–10 kPa), while maintaining sensitivities of 49 kPa⁻¹ and 11 kPa⁻¹, respectively, over the extended pressure ranges of 10–100 kPa and 100–200 kPa. These comparisons show that multilayer PGC structures provide not only high initial sensitivity but also a wide linear sensing range, thereby alleviating the sensitivity–operating-range trade-off observed in many previously reported pressure sensors.

**Figure 2**. Comparison of response time and repeatability durability between the PGC pressure sensor developed in this study and the reported pressure sensors

The response time and pressure cycle count of the reported pressure sensors (References S1–S5 and S9–S12) were compared with those of the PGC sensors used in this study. This PGC sensor demonstrated excellent performance by maintaining stable detection performance over more than 100,000 load cycles while exhibiting a response time of 69.3 ms, combining both fast response speed and long-term repeat durability.

**Supplementary references**

[1] Ma Y, Cheng Y, Wang J, Fu S, Zhou M, Yang Y, Li B, Nan CW, *InfoMat* **2022**, 4, e12328.

[2] Huang Y, Liu B, Zhang W, Qu G, Jin S, Li X, Nie Z, Zhou H, *npj Flex. Electron.* **2022**, 6, 92.

[3] Wei Y, Shi X, Yao Z, Zhi J, Hu L, Yan R, Shi C, Yu HD, Huang W, *npj Flex. Electron.* **2023**, 7, 13.

[4] Xie Y, Cheng Y, Ma Y, Wang J, Zou J, Wu H, Yue Y, Li B, Gao Y, Zhang X, Nan CW, *Adv. Sci.* **2023**, 10, 2205303.

[5] Park M, Yang W, Kim JW, Choi Y, Kim S, Lee Y, Kim DS, Kim J, Lim DK, Ha JS, *Adv. Funct. Mater.* **2024**, 34, 2402508.

[6] Cao M, Leng M, Pan W, Wang Y, Tan S, Jiao Y, Yu S, Fan S, Xu T, Liu T, Li L, Su J, *Nano Energy* **2023**, 112, 108492.

[7] Li L, Liu Y, Wang Z, Wang D, Yang K, Hu H, Qi S, Fang L, Han L, Zhang R, *Chem. Eng. J.* **2024**, 498, 154355.

[8] Zhang M, Gong S, Hakobyan K, Gao Z, Shao Z, Peng S, Wu S, Hao X, Jiang Z, Wong EH, Liang K, Wang CH, Cheng W, Xu J, Adv. Sci. 2024, 11, 2309006.

[9] Chen X, Luo Y, Chen Y, Li S, Deng S, Wang B, Zhang Q, Li X, Li X, Wang C, He J, Tian H, Shao J, *Adv. Sci.* **2024**, 11, 2408082.

[10] Li J, Li N, Zheng Y, Lou D, Jiang Y, Jiang J, Xu Q, Yang J, Sun Y, Pan C, Wang J, Peng Z, Zheng Z, Liu W, *Adv. Sci.* **2022**, 9, 2201912.

[11] Wang H, Dou X, Wang Z, Liu Z, Ye Q, Guo R, Zhou F, *Small* **2023**, 19, 2303234.

[12] Xia X, Xiang Z, Gao Z, Hu S, Zhang W, Long R, Du Y, Liu Y, Wu Y, Li W, Shang J, Li RW, *Adv. Sci.* **2024**, 11, 2304409.
