# Supplementary material for: High‐Performance Piezoresistive Sensors via Pd/Sn‐Free Graphene‐Catalyzed Metallization of Thermally Repairable Microfiber Scaffolds
Source: Adv Sci (Weinh). 2026 Sep 16:e77794. Online ahead of print. doi: 10.1002/advs.77794 (PMC13583221; doi:10.1002/advs.77794)
Supplement: Supplementary file 2 [file ADVS-9999-e77794-s004.docx]

Copyright WILEY-VCH Verlag GmbH & Co. KGaA, 69469 Weinheim, Germany, 2013.

Supporting Information

High-Performance Piezoresistive Sensors via Pd/Sn-Free Graphene-Catalyzed Metallization of Thermally Repairable Microfiber Scaffolds

*ByungJun Kim, Su Bin Choi, Yeonwook Jeong, Jeong-Won Yoon,* Seung-Boo Jung, Jong-Woong Kim**

**Characterization**

The microstructure and elemental composition of the fiber matrix were characterized by field emission scanning electron microscopy (FE SEM; Hitachi High Tech) equipped with an energy dispersive X ray spectroscopy (EDS) detector. Low magnification surface morphology was examined using an optical microscope (DM2700M, Leica). Electrical performance was evaluated under constant voltage using a Keithley 2461 SourceMeter, while sensor element resistance was measured with an LCR 6100 meter (GW Instek). Tensile properties of the fiber mat were assessed on a Shimadzu AGXTM V2 universal testing machine. Long term operational stability was determined via cyclic loading tests using a JIPT 120 tester (Junil Tech). Infrared transmittance spectra of the polymer components were acquired with an IRTracer 100 Fourier-transform infrared (FTIR) spectrophotometer (Shimadzu). Ultrasonic cleaning (Branson CPX1800H E) was employed to remove loosely bound graphene flakes, and Ag NWs were deposited on the PCL support via spray coating with a RichPen Apollo 113C airbrush.

**Figure S1.** Optical images of the poly(ε-caprolactone) (PCL) coating process on a glass substrate: (a) immediately after spin-coating of the PCL solution, prior to solvent evaporation, and (b) after complete solvent evaporation, revealing the formation of a uniform solid PCL film.

The images illustrate the transition from a liquid-phase polymer dispersion to a dry, continuous film. This step is critical for creating a stable base layer for mechanical support and adhesion in subsequent fiber assembly and sensor fabrication.

**Figure S2.** (a) Optical image of a PCL film scratched using a tape blade on a glass substrate after thermal curing at 80 °C, and (b) image of the same region after cooling for 5 min at ambient conditions.

These images highlight the thermal softening behavior of PCL, which facilitates localized deformation and groove formation upon mechanical scratching. The retention of the groove structure after cooling indicates thermal setting of the polymer surface, enabling its use as a mechanical anchoring or alignment guide during subsequent sensor assembly steps.

**Figure S3.** (a) Photograph of a 10 × 10 mm PCL/Graphene/Cu(PGC) fiber sheet used as the sensing element. (b) Zigzag-shaped PCL support fabricated by scoring a thermally cured PCL film using a serrated tape-cutting blade to enable folding and mechanical flexibility. (c) PGC fiber sheet adhered to the PCL support using a low-concentration PCL solution as a thermoplastic adhesive. (d) A five-layered PGC sensor structure mechanically reinforced and stabilized with two PCL supports. (e) Copper tape and conductive silver paste used for attaching external electrodes to the multilayer sensor assembly. (f) Experimental setup for evaluating pressure sensitivity by applying a defined load while biasing the sensor.

This sequence illustrates the complete device assembly workflow, from material preparation to final integration with electrical contacts. The use of thermally moldable PCL and layered architecture allows modular customization of sensor thickness, contact fidelity, and mechanical stability for precision pressure transduction.

**Figure S4.** Digital photographs of PCL support surfaces coated with silver nanowire (AgNW) solutions of varying concentrations: (a) 0.1 wt%, (b) 0.2 wt%, (c) 0.3 wt%, (d) 0.4 wt%, and (e) 0.5 wt%.

The progression demonstrates the tunability of surface coverage and optical characteristics as a function of AgNW concentration. Increasing concentration results in more continuous and reflective conductive networks, which directly impact the baseline conductivity and transparency of the sensor’s electrode layer.

**Figure S5.** Enlarged optical images of spray-coated PCL support surfaces as a function of AgNW concentration: (a) pristine PCL sheet, (b) 0.1 wt% AgNW, (c) 0.2 wt%, (d) 0.3 wt%, (e) 0.4 wt%, and (f) 0.5 wt%.

These high-magnification views highlight the evolution of nanowire distribution and surface coverage, which influence the trade-off between electrical conductivity and transparency. At higher concentrations, AgNWs form interconnected conductive pathways that enhance electron transport but may increase optical haze—factors critical for optimizing electrode performance in pressure-sensing applications.

**Figure S6.** Comparison of the electrical resistance of the PCL support layer as a function of AgNW solution concentration: 0.1, 0.2, 0.3, 0.4, and 0.5 wt%.

As the AgNW concentration increases, the sheet resistance of the support layer decreases markedly due to the formation of more densely connected percolation networks. This trend reflects a typical percolation threshold behavior, where the establishment of continuous conductive paths enhances electrical performance. Optimizing this balance is crucial for minimizing energy loss while preserving mechanical flexibility and structural integrity.

**Figure S7.** Optical images of fiber matrices at different stages of conductive coating: (a) pristine electrospun PCL fiber sheet, (b) PCL fiber sheet coated with graphene via dip-coating and mild drying, and (c) PCL/graphene fiber sheet after electroless copper plating.

The progressive structural evolution demonstrates the sequential integration of functional materials. The graphene layer provides a conformal, conductive interface while preserving fiber morphology, and the subsequent copper plating forms a continuous metallic shell. This hierarchical construction is key to achieving enhanced electrical conductivity and mechanical robustness without compromising the fiber network’s porosity.

**Figure S8.** EDS carbon mapping images of graphene-coated PCL fiber mats: (a) before and (b) after ultrasonication treatment.

The elemental carbon distribution highlights the impact of ultrasonication on graphene flake dispersion across the fiber surface. Prior to ultrasonication, agglomerated graphene regions dominate, leading to non-uniform surface coverage. Post-treatment, a more homogeneous carbon signal indicates improved dispersion and intimate graphene–fiber interfacial contact—critical for uniform copper nucleation during subsequent electroless plating.

**Figure S9.** EDS copper (Cu) elemental mapping of PGC conductive fibers.

The mapping image illustrates the uniform distribution of copper throughout the fiber network, indicating successful and conformal electroless plating on the graphene-coated PCL substrate. The continuous and homogeneous presence of Cu highlights the effectiveness of the graphene interlayer in promoting even nucleation and growth, which is critical for maintaining reliable electrical conductivity and mechanical stability across the sensing matrix.

**Figure S10.** FTIR spectra of pristine PCL, PCL/Graphene, and PGC composite fiber mats.

Characteristic absorption bands of PCL are evident at 2940 cm^-1^ (C–H asymmetric stretching), 1735 cm^-1^ (carbonyl C=O stretching), and 1235 cm^-1^ (C–O–C stretching). With the introduction of graphene and subsequent electroless copper plating, the intensity and resolution of these peaks progressively diminish.

**Figure S11**. Comparative analysis of Cu deposition behavior on PCL fiber mats with and without graphene or Pd/Sn activation, and mechanical stability of the graphene-mediated Cu coating. (a) Optical microscopy (OM) images of PCL/Cu, PCL/Pd-Sn/Cu, PCL/graphene (PG), and PCL/graphene/Cu (PGC) fiber mats. (b) Surface SEM image of the PCL/Cu sample. (c) Surface SEM image of the PCL/Pd-Sn/Cu sample. (d) Surface SEM image of the PCL/graphene/Cu sample at the early stage of Cu nucleation, showing the initial formation of Cu deposits on the graphene-coated fibers. (e) Surface SEM image of the fully developed PGC sample, showing a continuous Cu-coated fibrous network. (f) Electrical resistance comparison of PCL/Cu, PCL/Pd-Sn/Cu, and PGC samples, demonstrating the markedly improved conductivity of the graphene-mediated Cu coating. (g) Surface SEM image of the PGC sample after 10 tape-peeling cycles. (h) Relative resistance change of the PGC sample as a function of tape-peeling cycle number, indicating the electrical stability of the Cu coating under repeated peeling. (i) Surface SEM image of the PGC sensor after 100,000 loading cycles. (j) SEM-EDS elemental mapping and compositional analysis of the PGC sensor surface after 100,000 loading cycles, confirming the retention of the Cu coating on the fibrous network. (k) Cross-sectional/interface SEM image of the PGC sensor after 100,000 loading cycles. (l) SEM-EDS elemental mapping and compositional analysis of the interfacial region after 100,000 loading cycles, demonstrating the preserved Cu-containing conductive layer and interfacial integrity.

This figure compares the Cu deposition behavior on pure PCL, Pd/Sn surface-treated PCL, and graphene-coated PCL fibers. The graphene layer plays a crucial role in promoting continuous Cu growth. In pure PCL and Pd/Sn-activated PCL, Cu deposition was not uniform due to the hydrophobic characteristic of the fiber surface. Graphene bonded to the polymer fiber surface formed a more uniform Cu plating layer. After repeated tape peeling tests, interfacial SEM/EDS analysis revealed that the graphene-mediated Cu layer was stably attached, and no interfacial delamination occurred even after repeated mechanical deformation.

**Figure S12.** Optical images of pristine PCL fiber mats subjected to thermal treatment at increasing temperatures. (a) Unheated reference sample, (b) after 10 min of heating at 40 °C, (c) at 50 °C, (d) at 60 °C, (e) at 70 °C, and (f) at 80 °C.

As the temperature approaches and exceeds the melting point of PCL (~60 °C), progressive softening and fiber fusion become evident, leading to loss of structural integrity and porosity. These observations highlight the thermal response of the PCL matrix and serve as a reference for evaluating the morphological stability and recovery behavior of the composite systems.

**Figure S13.** Optical images of PCL/Graphene composite fiber mats before and after thermal treatment at various temperatures. (a) Unheated PCL/Graphene sample, (b) after 10 min at 60 °C, (c) 70 °C, (d) 80 °C, (e) 100 °C, (f) 120 °C, (g) 140 °C, and (h) 160 °C.

With increasing temperature, the composite exhibits gradual morphological changes, including localized fiber swelling, fusion, and eventual distortion of the porous network. The graphene coating serves as a thermal barrier and mechanical scaffold, helping to preserve the mat's macrostructure even at elevated temperatures. These thermally induced transitions provide insight into the stability limits and deformation behavior of the graphene-enhanced PCL matrix.

**Figure S14.** Optical images of PGC composite fibers before and after thermal exposure at various temperatures. (a) As-prepared PGC fiber mat prior to heating, (b) after 10 min at 60 °C, (c) 80 °C, (d) 100 °C, (e) 120 °C, (f) 140 °C, (g) 160 °C, and (h) 180 °C.

The fiber morphology remains largely intact across the tested temperature range, indicating exceptional thermal stability imparted by the conformal copper overlayer. While minor surface oxidation or color changes may occur at higher temperatures, the structural integrity of the mat is preserved even at 180 °C. This thermomechanical resilience demonstrates the synergistic protective effect of the graphene and Cu coatings, which restrict polymer flow and deformation under thermal stress, thereby extending the operational temperature window of the sensor platform.

**Figure S15.** Resistance variation of the PGC matrix during thermal heating and cooling cycles

The PGC sample showed a reversible increase in resistance when heated to 200°C. Upon stopping the heat heating and cooling to 0°C, it recovered to its initial resistance. This result shows the thermal robustness and reversible electrical response of the PGC conductive network in high temperature cycling.

**Figure S16.** (a) Final tensile strength (UTS) recovery of PCL, PG, and PGC after repaired. (b) Elongation recovery of PCL, PG, and PGC after repaired.

This figure summarizes the mechanical recovery of PCL, PG, and PGC after one recovery cycle. Both the UTS recovery and elongation recovery values show that all samples regained a substantial fraction of their original mechanical performance after recovering, while the PGC sample exhibited the highest recovery among the three groups. These results indicate that the graphene/Cu-modified fibrous network more effectively retains mechanical integrity after thermal recovered.

**Figure S17.** Thermally assisted functional recovery of the fabricated PGC fiber matrix: (a) Digital and (b) optical images capturing the damage-and-recovery process of the PGC fiber network. (a-1, b-1) Intact fiber matrix before cutting, (a-2, b-2) fiber matrix after partial cutting, (a-3, b-3) after thermal repair at 80 °C for 15 min, and (a-4, b-4) mechanical stretching of the thermally repaired region. (c) Electrical resistance variations during repeated cut-and-heal cycles. (d) Comparison of tensile strength between pristine and repaired PGC fiber matrices.

This figure shows the heat-assisted recovery behavior of the PGC fiber matrix after damage. A portion of the PGC was cut using a Dorco knife and the cut portion was heat-treated at 80°C for 15 minutes to recombine. It was visually confirmed that the conductive path was restored and mechanically bound.

F**igure S18.** Quantitative analysis of the proposed layer-by-layer contact mechanism in multilayer PGC sensors. (a) Schematic illustration of conductive junction formation in 2-5 layers PGC sensors under compression, with the corresponding pressure-response curves. (b) Pressure-dependent thickness variation of multilayer PGC sensors. Insets show representative side-view images before and during pressing. (c) Pressure-dependent compressive strain calculated from the measured thickness values.

This figure quantitatively shows the proposed layer-by-layer contact mechanism in multilayer PGC sensors. As the number of stacked PGC layers increases, a larger number of conductive paths increase under compression, which expands the pressure sensing range. The measured thickness reduction and the resulting compression deformation show that as the layer trough increases, the pressure perception range of multilayer PGC sensors increases.

**Figure S19.** Experimental validation of real-time pressure response using a pushing tester system. (a) Photograph showing the experimental setup in which a mechanical pushing tester applies a controlled compressive force to the PGC fiber matrix pressure sensor. (b) Screen capture of the SourceMeter display indicating a stable current output under an applied pressure of 60 kPa.

The steady-state current response confirms the sensor’s high measurement fidelity and repeatability under static loading conditions. This setup demonstrates the sensor’s ability to deliver reliable electrical output during precise pressure application, essential for calibration and performance characterization in practical applications.

**Figure S20.** High-temperature durability assessment of the PGC-based pressure sensor.

The thermal resilience of the PGC fiber matrix was systematically examined by preheating samples at various temperatures—room temperature (RT), 60 °C, 100 °C, 140 °C, and 180 °C—for 15 min, followed by ambient cooling for 30 min. After thermal conditioning, the fiber matrices were assembled into pressure sensors and subjected to cyclic loading (1 Hz, 100 kPa) to evaluate electromechanical stability. Across all treatment conditions, the sensors displayed consistent and stable current outputs, indicating robust retention of sensing capability. Notably, the sensor exposed to 180 °C exhibited only a slight decrease (2.63%) in output current relative to the untreated control, underscoring the excellent thermal tolerance of the hierarchical PGC structure. This performance highlights the sensor’s potential for deployment in environments involving elevated temperatures without significant compromise in functionality.

**Figure S21.** Electrical current response under repeated mechanical loading at various pressures.

The PGC-based pressure sensor was subjected to five repeated loading–unloading cycles at each of the following pressure levels: 0.098 kPa, 5 kPa, 25 kPa, 50 kPa, 100 kPa, and 200 kPa. The recorded current responses demonstrate excellent repeatability and minimal drift across all pressure conditions, confirming the sensor's stability and consistent electromechanical performance under both low and high loads.

**Figure S22.** Extended constant-pressure hold test and quantified signal drift of the PGC sensor under static loading. (a) Current of the pressure sensor when constant pressures of 20, 50, 100, and 200 kPa are applied for approximately 8000 seconds. (b) Initial current, final current, normalized drift, and drift rate data for each pressure condition.

The sensor exhibits signal stability when a constant load is applied for 2.2 hours, and drift decreases as the applied pressure increases.

**Figure S23.** Electrical and mechanical stability test of multilayer PGC pressure sensor under bending conditions. (a) Image of the sensor in flat condition, bending radius 10mm, 5mm, and 3mm.(b) Current change with pressure under different bending radius. (c) Current response under repeated loading at 50 kPa for different bending radius 10, 5, and 3 mm show stable outputs under curved conditions. (d) Current signal value of the sensor during 500 repetitions of bending & relaxation cycles in a bending radius of 10mm (e) Peak response retention of the sensor during 500 bending/releasing cycles at a bending radius of 10 mm.

**Figure S23** shows the electrical and mechanical stability of the multilayer PGC sensor under bending deformation. The sensor maintained stable current change even with bending radius up to 3 mm. In addition, no degradation in signal output was observed during about 500 repetitive bending & relaxation cycles, confirming the mechanical stability of the multilayer structure.

**Figure S24.** Evaluation of the Electrical Stability of Multilayer PGC Pressure Sensor with Sheared Movement during Load Application. (a) Image of the shear test showing the sensor under constant preload conditions and after applying lateral shear movement. (b) Current change in sensor when shear displacement is 0 to 3 mm at constant pressure of 50 kPa. (c) Peak response retention of the PGC sensor during repeated shear cycles at different shear displacements.

It shows the results of the electrical stability evaluation of the PGC sensor according to the lateral shear movement of the object during load application. Even in shear displacement of up to 3 mm under a constant load of 50 kPa, the sensor maintained a stable current output and a consistent response throughout the repetitive cycle, although some noise occurred.

**Figure S25**. Reproducibility evaluation of independently fabricated PGC sensors. (a) Histogram of the initial resistance values measured from 57 PGC sensors fabricated across independent batches (b) Current change graph of 5 independently fabricated PGC pressure sensors

The histogram of initial resistance values obtained from 57 independently fabricated sensors shows a relatively narrow distribution, indicating limited device-to-device variation in the initial electrical state. In addition, five representative independently fabricated sensors exhibited highly similar pressure-dependent current responses over the entire pressure range.

**Figure S26.** Objects with different masses used to evaluate the performance of the pressure sensor array: (a) a pinecone (4.67 g), (b) a steel weight (10 g), and (c) a paper plane (1.46 g).

These objects, varying in shape, contact area, and mass, were chosen to demonstrate the array’s ability to resolve diverse pressure profiles and reliably detect both lightweight and irregularly shaped items—critical for practical applications in wearable electronics and tactile interfaces.

**Figure S27.** Top and side-view schematics of the fabricated 6×6 pressure sensor array structure.

Illustrated schematics showing (top) the planar layout and (side) the cross-sectional configuration of the 6×6 pressure sensor array. Each sensing unit is composed of a layered PGC matrix positioned between orthogonally aligned electrode lines, enabling localized pressure detection.

**Figure S28.** Optical image of a pinecone placed on the surface of the sensor array.

The image illustrates the physical contact between a pinecone and the 6×6 pressure sensor array. Notably, only a portion of the pinecone makes direct contact with the array surface due to its irregular geometry and non-uniform base.

**Figure S29.** Stepwise fabrication process of a paper plane and multi-angle photograph of the final object.

This figure presents the step-by-step folding process for constructing a paper plane, followed by a photograph of the completed plane taken from an alternative angle. The image reveals that both the central fuselage and the outer wing regions of the paper plane come into contact with the pressure sensor array.

**Figure S30**. Demonstration of self-powered LED switching using a TENG-driven PGC sensor array. (a) Schematic diagram of the application circuit. The output of the triboelectric nanogenerator (TENG) is rectified through a bridge rectifier and applied to a PGC sensor array acting as a pressure-sensing switch to control the LED. (b) Photograph of the fabricated PGC sensor array integrated into the switching circuit. (c) Photograph of the device with no pressure applied. It shows the LED turned off. (d) Photograph of the device with pressure applied.

The PGC sensor array closes the circuit, allowing current to flow and the LED to light up. These results demonstrate that the PGC sensor array is feasible as a pressure-responsive switching device for self-powered interactive electronic applications.

**Supplementary Video 1:** Repetitive application of a constant 30 kPa pressure on the PGC-based pressure sensor.

This video demonstrates the real-time electromechanical response of the PGC pressure sensor under repeated mechanical loading at a constant pressure level of 30 kPa. The sensor exhibits consistent and stable current output across multiple loading cycles, confirming its excellent repeatability, durability, and structural resilience. This performance highlights the sensor’s suitability for practical applications requiring reliable detection of medium-range pressures over extended operational periods.

**Supplementary Video 2:** Pressure-triggered LED switching demonstration using the PGC sensor array.

This video presents a proof-of-concept demonstration of a PGC sensor array integrated with a simple LED circuit. When external pressure is applied to the sensor array, the device acts as a pressure-triggered switch, turning on the connected LED in real time. When the pressure is released, the LED turns off. This demonstration highlights the feasibility of integrating the fabricated sensor array with external electronic devices for interactive pressure-responsive applications.
